# Conditioned Media‐Derived Tumor Extracellular Vesicles: Bridging Molecular Insights and Therapeutic Applications in Oncology

**DOI:** 10.1002/wnan.70077

**Published:** 2026-08-20

**Authors:** Alaya Alkaabi, Dana Nasrallah, Roberta Giordo, Gheyath K. Nasrallah, Hatem Zayed, Gianfranco Pintus

**Affiliations:** ^1^ Department of Biomedical Sciences, College of Health Sciences, QU Health Qatar University Doha Qatar; ^2^ Department of Human Science for Promotion of Quality of Life University San Raffaele Rome Italy; ^3^ Department of Biomedical Sciences University of Sassari Sassari Italy

**Keywords:** conditioned media, EV‐associated biomarkers, EV‐based therapeutics, EV‐enriched preparations, tumor‐derived extracellular vesicles

## Abstract

Tumor‐derived extracellular vesicles (EVs) are major mediators of oncogenic intercellular communication. However, many studies using conditioned media (CM) recover operationally defined EV‐ or small EV‐enriched fractions rather than biogenesis‐proven exosomes. This review synthesizes evidence from CM‐derived tumor EV studies and aligns their biological interpretation with current EV nomenclature, characterization, and reporting principles. We first discuss exosome biogenesis as a defined endosomal pathway and then examine how CM collection, serum handling, culture format, oxygen tension, conditioning interval, isolation strategy, storage, and co‐isolated material shape EV yield, cargo composition, and apparent biological activity. Mechanistically, CM‐based studies have clarified how tumor‐derived EV‐enriched preparations can support pre‐metastatic niche formation, immune evasion through PD‐L1 and regulatory RNA cargo, stromal and endothelial remodeling, angiogenesis, and therapy resistance. We also evaluate EV‐associated miRNA and protein biomarkers as candidate liquid‐biopsy analytes, emphasizing that their diagnostic value remains assay‐, cohort‐, and workflow‐dependent. Therapeutically, the review distinguishes mechanisms inferred from CM‐derived tumor EV studies from engineered EV platforms for drug, RNA, cytokine, vaccine, or immune‐agonist delivery. Across these areas, we emphasize that robust EV‐specific biological interpretations require complementary and methodologically independent characterization, purity and co‐isolate assessment, EV‐depleted CM, add‐back or rescue designs, cargo perturbation, enzymatic controls where appropriate, and dose‐normalized functional assays. Advances in microfluidics, immunoaffinity capture, single‐vesicle analysis, and multi‐omics profiling are improving resolution, but translation will depend on standardized workflows, product‐ or biomarker‐specific validation, scalability, potency metrics, safety, regulatory‐grade quality control, and more explicit integration with in vivo and clinically annotated datasets.

This article is categorized under:

Nanotechnology Approaches to Biology > Nanoscale Systems in Biology
Diagnostic Tools > Diagnostic Nanodevices
Therapeutic Approaches and Drug Discovery > Nanomedicine for Oncologic Disease

## Graphical abstract

CM‐based workflow for enriching tumor‐derived EV‐containing fractions and testing their biological effects. Tumor cells cultured under defined conditions, typically in EV‐depleted serum‐supplemented medium or other controlled culture systems, release extracellular vesicles (EVs) together with soluble and non‐vesicular extracellular components into conditioned medium (CM). After CM collection and pre‐processing, EV‐containing fractions can be enriched using representative workflows such as ultracentrifugation. The resulting EV‐enriched preparations can then be applied to recipient cells in vitro or administered in vivo to investigate tumor EV‐associated biological effects under defined experimental conditions. This workflow highlights the value of CM systems for mechanistic studies and defines the recovered material as operationally EV‐enriched unless vesicle subtype, purity, and biogenesis are demonstrated by appropriate characterization and controls.

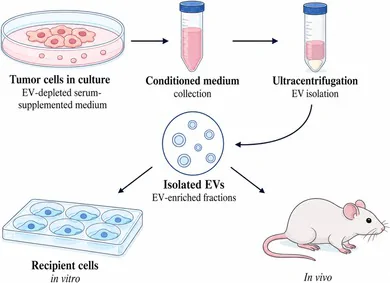

## 1 Introduction

Extracellular vesicles (EVs) are lipid bilayer‐delimited particles released by cells and unable to replicate independently (Thery et al. 2018; Welsh et al. 2024). Within this heterogeneous family, small EV‐enriched populations can overlap in size with vesicles historically described as exosomes, often within an approximate 50–150 nm range (Coughlan et al. 2020); however, size alone does not establish endosomal origin. EVs participate in intercellular communication and have been investigated across a broad range of pathological settings, including metabolic and diabetes‐related complications, cardiovascular disease, cardiomyopathies, neurological disorders, and cancer‐associated regulatory RNA biology (Abbas et al. 2024; Almaghrbi et al. 2023; FawzuAmeer et al. 2024; Gopikrishnan et al. 2023; Wehbe et al. 2025; Zhang et al. 2019). Within this broader pathophysiological framework, their involvement in cancer has attracted particular attention, as tumor‐associated EVs can contribute to tumor–stromal communication, microenvironmental remodeling, immune modulation, metastatic niche formation, therapy resistance, and biomarker discovery (Greening et al. 2025; Hoshino et al. 2015; Kalluri et al. 2025).

In keeping with MISEV2018 and MISEV2023 recommendations, the term EVs is used as the generic term for “extracellular vesicles”, whereas “exosomes” is reserved for contexts in which endosomal biogenesis is specifically discussed or supported (Thery et al. 2018; Welsh et al. 2024). This distinction is particularly important in conditioned media (CM)‐based studies, where many workflows enrich heterogeneous EV or small EV populations but, by themselves, do not establish vesicle origin. Therefore, throughout the manuscript, “tumor‐derived EVs” is used as the default descriptor unless a cited study specifically demonstrates, or explicitly discusses, endosome‐derived exosomes (Thery et al. 2018; Welsh et al. 2024).

When the term exosome is biogenetically justified, it refers to a biogenesis‐defined EV subset generated through the endosomal pathway: endocytosis gives rise to early endosomes, late endosomes, and multivesicular bodies (MVBs) containing intraluminal vesicles (ILVs), which can be released extracellularly after MVB fusion with the plasma membrane (Han et al. 2022; Mashouri et al. 2019; Arya et al. 2024). Markers and molecules commonly associated with EV‐enriched preparations, including CD9, CD63, CD81, TSG101, ALIX, and other pathway‐related components, are useful for characterization but should not be interpreted as stand‐alone proof of purity, subtype identity, or exosomal origin (Lai et al. 2022). This interpretation is consistent with current MISEV guidance (Welsh et al. 2024).

EV release and cargo composition are sensitive to cell state and experimental context, including stress, hypoxia, metabolic perturbation, inflammatory cues, and culture conditions (Abramowicz et al. 2018; Shekari et al. 2023). This context sensitivity is central to CM‐based cancer studies, given that the observed EV yield, molecular profile, and biological activity may reflect both tumor‐intrinsic programs and pre‐analytical variables introduced during cell culture and sample handling. For this reason, culture and pre‐analytical variables in CM studies should be treated as part of the experimental design, not as neutral background conditions (Shekari et al. 2023; Welsh et al. 2024).

Tumor cells can release abundant EVs that communicate with neighboring stromal, endothelial, and immune cells as well as distant tissues (Dai et al. 2020; Ruivo et al. 2017). These tumor‐derived EVs carry proteins, lipids, and nucleic acids that partially reflect the molecular landscape of the parent tumor cells (Dai et al. 2020). By transferring these cargos, tumor‐derived EVs can modulate recipient‐cell behavior and contribute to cancer‐associated processes such as angiogenesis, invasion, metastasis, immune evasion, and therapy resistance (Tang et al. 2025; Wu, Han, et al. 2024).

Advances in the isolation and analysis of EVs from CM have improved the mechanistic study of tumor cell‐derived vesicle communication (Mukerjee et al. 2025; Singh et al. 2024). CM is valuable since donor cell identity, culture conditions, and experimental perturbations can be controlled more tightly than in most biofluids (Shekari et al. 2023). However, CM should not be viewed as intrinsically standardized: applying the same isolation protocol does not necessarily generate comparable EV populations, because serum‐depletion strategy, cell density, and confluence, culture format, conditioning interval, mycoplasma status, and CM collection, storage, or freeze–thaw handling can substantially alter EV yield, cargo composition, and apparent bioactivity (Shekari et al. 2023). Accordingly, the major value of CM lies not in automatic reproducibility, but in the possibility of designing rigorously controlled mechanistic experiments in which these variables are explicitly reported and experimentally controlled (Shekari et al. 2023). In serum‐containing workflows, residual serum‐derived EVs, proteins, and lipoprotein‐associated material may also contribute to the recovered preparation; therefore, explicit reporting of serum source and depletion strategy is required (Pham et al. 2021; Shekari et al. 2023; Welsh et al. 2024).

The available literature on CM‐derived tumor EVs provides an integrated view of their mechanistic roles and translational implications in oncology. Given that many cited studies use exosome terminology without definitive proof of endosomal origin, the term is interpreted operationally when the vesicle subtype is not experimentally established (Thery et al. 2018; Welsh et al. 2024). In the therapeutic applications discussion, we distinguish mechanisms deduced from CM‐derived tumor EV studies from those in the broader field of engineered EV‐ or exosome‐based platforms. Early clinical work using dendritic cell‐derived exosomes as cancer vaccines illustrates the translational interest in this area (Escudier et al. 2005), while later reviews have emphasized both the therapeutic potential of EV‐based approaches and the unresolved challenges in clinical development (Di Bella 2022; Rezaie et al. 2022). A recent cancer‐focused synthesis also characterizes EVs as both diagnostic and therapeutic tools, while highlighting ongoing translational barriers (Greening et al. 2025). In this context, therapeutic translation is considered product‐ and platform‐specific, necessitating clear source definition, process control, dose metrics, potency assessments, and quality attributes (Xu, Jin, et al. 2025).

Within this context, CM‐based studies have provided particularly useful mechanistic insight into tumor‐derived EV biology in cancer. In this review, we emphasize how CM systems have enabled mechanistic dissection of metastasis, immune evasion, stromal reprogramming, and therapeutic resistance. We also examine the methodological caveats that condition interpretation and translation, including EV nomenclature, culture and pre‐analytical variability, characterization, purity, reproducibility, functional causality controls, and EV dose normalization.

## 2 EV Biogenesis and Isolation From CM

Interpretation of conditioned medium (CM)‐derived tumor EV studies requires a clear conceptual and methodological distinction between biogenesis‐defined exosomes, operational EV‐enriched preparations, isolation workflows, co‐isolates, and functional controls. We first clarify exosome biogenesis as a biogenesis‐defined EV pathway, emphasizing that most CM workflows recover operationally defined EV‐ or small EV‐enriched fractions, not biogenesis‐proven exosomes (Thery et al. 2018; Welsh et al. 2024). We then discuss how CM collection, serum handling, cell‐culture conditions, isolation strategy, and downstream processing influence EV yield, cargo composition, co‐isolate burden, and apparent biological activity (Shekari et al. 2023; Welsh et al. 2024). Finally, we examine the major enrichment approaches used for CM‐derived tumor EVs, including ultracentrifugation, ultrafiltration, size‐exclusion chromatography, and immunoaffinity‐based methods, before addressing characterization, purity, reproducibility, functional causality controls, and EV dose normalization. This distinction separates mechanistic insight from methodological artifact and defines CM‐derived EV preparations as experimentally informative but operationally defined materials unless vesicle subtype, purity, and functional specificity are supported by appropriate evidence.

### 2.1 Exosome Biogenesis as a Biogenesis‐Defined EV Pathway

Considering that “exosome” refers specifically to an EV subtype generated through the endosomal pathway (Thery et al. 2018; Welsh et al. 2024), exosome biogenesis provides an essential mechanistic reference point for interpreting CM‐derived small EV‐enriched preparations. However, most conditioned‐media studies operationally isolate heterogeneous EV or small‐EV‐enriched fractions, rather than exosomes (Shekari et al. 2023; Thery et al. 2018; Welsh et al. 2024). Exosome biogenesis therefore provides a mechanistic framework, but it does not imply that all small EV fractions recovered from CM are exosomes. Recent reviews further emphasize that EV biogenesis is pathway‐diverse and context‐dependent, reinforcing the need to avoid assigning exosomal origin solely based on size, density, or marker detection (Arya et al. 2024; Yu et al. 2023).

**FIGURE 1:**
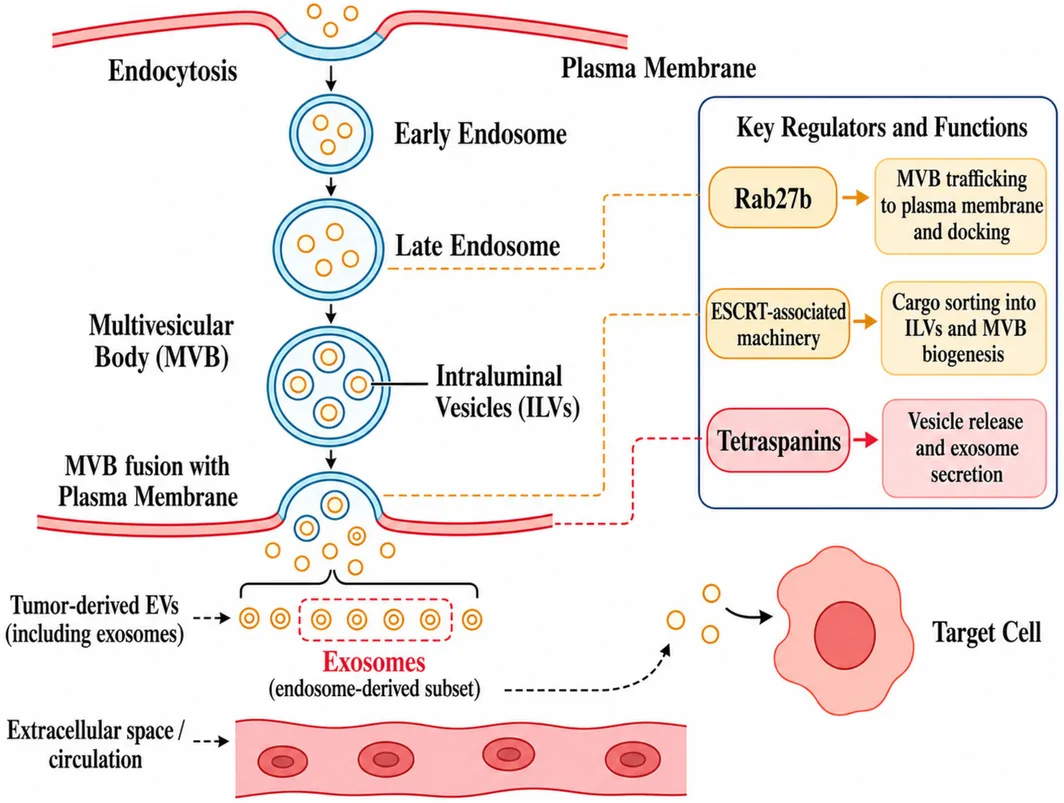
Biogenesis and secretion pathway of tumor‐derived EVs, with emphasis on endosome‐derived exosome formation. The schematic illustrates the canonical endosomal pathway through which exosomes are generated as a biogenesis‐defined subset of tumor‐derived extracellular vesicles (EVs). Endocytosis at the plasma membrane leads to the formation of early endosomes, which mature into late endosomes and multivesicular bodies (MVBs). Within MVBs, inward budding generates intraluminal vesicles (ILVs), which can be released extracellularly as exosomes following MVB fusion with the plasma membrane. The figure highlights representative regulatory components, including Rab27‐family proteins involved in MVB trafficking and docking, ESCRT‐associated machinery involved in cargo sorting and MVB biogenesis, and tetraspanins associated with membrane organization and vesicle release. Once released into the extracellular space, tumor‐derived EVs can interact with recipient cells and contribute to intercellular communication processes involved in tumor progression. This figure provides a conceptual biogenesis framework and does not imply that all EV‐enriched fractions isolated from conditioned media are exosomes; such fractions remain operationally defined unless endosomal origin is specifically demonstrated. The endosomal sequence shown here is supported by mechanistic biogenesis reviews (Han et al. 2022; Mashouri et al. 2019), whereas the operational caveat for CM‐derived EV‐enriched fractions follows MISEV guidance (Thery et al. 2018; Welsh et al. 2024). The Rab/ESCRT/tetraspanin elements are representative regulatory modules, not subtype‐defining markers (Han et al. 2022; Mashouri et al. 2019; Welsh et al. 2024).

In this biogenesis‐defined context, exosome formation involves an intracellular sequence initiated by endocytosis, in which invagination of the plasma membrane generates early endosomes that mature into late endosomes and multivesicular bodies (MVBs), which contain intraluminal vesicles (ILVs) (Han et al. 2022; Mashouri et al. 2019). When MVBs fuse with the plasma membrane, ILVs are released into the extracellular space as exosomes (Figure 1) (Han et al. 2022; Mashouri et al. 2019). This pathway is distinct from outward plasma‐membrane budding, apoptotic‐vesicle release, and other EV‐generating routes, which can produce vesicles with overlapping size and marker profiles but different biogenetic origins (Carvalho Ferraz et al. 2025; Yu et al. 2023).

Exosome biogenesis is regulated by both ESCRT‐dependent and ESCRT‐independent mechanisms. ESCRT‐associated machinery contributes to cargo sorting and ILV formation. In contrast, tetraspanins, lipid‐dependent pathways, ceramide‐related processes, and other membrane‐organizing systems can also influence cargo selection, ILV generation, and vesicle release (Arya et al. 2024; Carvalho Ferraz et al. 2025; Han et al. 2022; Mashouri et al. 2019). This mechanistic diversity is particularly relevant to cancer biology, as altered cargo sorting or vesicle release can alter the biological output of tumor‐derived EVs without necessarily indicating that the isolated vesicles are exclusively endosome‐derived exosomes.

Rab GTPases, ESCRT‐associated machinery, and tetraspanins contribute to MVB trafficking, cargo sorting, and vesicle release (Han et al. 2022; Zhang et al. 2022). In particular, Rab27a and Rab27b regulate distinct steps of exosome secretion, including MVB docking and fusion with the plasma membrane, whereas Rab31 marks an ESCRT‐independent exosome pathway by facilitating ILV formation and limiting MVB degradation, thereby promoting exosome output (Ostrowski et al. 2010; Wei et al. 2021). These molecules are therefore useful mechanistic regulators of EV production and cargo sorting, but their detection in isolated CM‐derived preparations should not be used as stand‐alone evidence of exosomal subtype identity (Thery et al. 2018; Welsh et al. 2024).

These pathways are responsive to cellular context and may be altered by inflammatory, metabolic, hypoxic, and neoplastic states (Han et al. 2022; Mashouri et al. 2019; Yeat and Chen 2025). For example, tumor necrosis factor‐alpha (TNF‐alpha) has been reported to stimulate EV release, including exosome secretion in defined contexts, supporting the broader concept that stress‐associated signaling can increase vesicle output in defined contexts (Han et al. 2022; Mashouri et al. 2019). In cancer, alterations in ESCRT‐related pathways, tetraspanins, and Rab GTPases can affect the abundance and molecular composition of released EVs (Han et al. 2022; Zhang et al. 2022), thereby supporting tumor‐promoting intercellular communication, including immune‐modulatory signaling (Rong et al. 2016). However, in most CM studies, the isolated fraction remains operationally defined unless endosomal origin has been specifically demonstrated (Shekari et al. 2023; Thery et al. 2018; Welsh et al. 2024).

### 2.2 Isolation of CM‐Derived Tumor EVs: Workflow, Variability, and Experimental Caveats

Isolation of tumor‐derived EV‐enriched fractions from conditioned medium (CM) is a central step in mechanistic in vitro studies. Typically, tumor cells are cultured under defined conditions, often in medium supplemented with EV‐depleted serum or, in selected settings, under serum‐free conditions for a limited interval, and are then allowed to condition the medium before CM is collected and processed to remove cells, debris, and larger extracellular particles (Mukerjee et al. 2025; Singh et al. 2024). CM is particularly useful since donor cell identity, culture conditions, and imposed perturbations can be controlled more tightly than in most biofluids, thereby facilitating the study of tumor‐derived EV cargo and function (Dowling and Clynes 2011). However, CM should be seen as an experimental system, not as an intrinsically standardized sample source. This distinction is important as cell culture‐conditioned medium‐derived EV studies require transparent reporting of the cellular source, culture conditions, and pre‐analytical variables used to generate the EV‐containing material (Shekari et al. 2023; Welsh et al. 2024).

Even when similar downstream isolation workflows are used, EV yield, cargo composition, co‐isolate burden, and apparent bioactivity can vary substantially according to serum‐depletion strategy, cell density and confluence, passage number, culture configuration, 2D versus 3D format, mono‐ versus co‐culture design, conditioning interval, oxygen tension, mycoplasma status, and CM collection, storage, or freeze–thaw handling (Shekari et al. 2023; Welsh et al. 2024). Recent work further supports this point by showing that 3D tumor‐organoid systems can generate EVs with altered cargo compared with conventional 2D culture (Schuster et al. 2024), and broader methodological reviews indicate that 3D culture can change EV yield, composition, morphology, and functional output relative to 2D platforms (Cook and Li 2025).

Serum handling is a particularly important pre‐analytical variable. Commercial and in‐house EV‐depleted serum preparations may retain different residual serum EV, protein, or lipoprotein backgrounds, and bovine or serum‐derived components can co‐isolate with cell‐derived EV preparations (Pham et al. 2021). Heat inactivation performed after EV depletion can also alter the proteome of subsequent cell‐derived EV preparations and introduce proteins that co‐isolate with EVs; this underscores the need to report both the serum source and the depletion/heat‐inactivation workflow (Urzi et al. 2024). Serum‐free conditioning can reduce serum‐derived contamination, but it may also induce stress responses that alter donor‐cell physiology and EV output; therefore, serum‐free intervals require justification and must be compatible with cell viability and the biological question under study. Serum‐depleted and serum‐free strategies should be reported and interpreted separately as distinct pre‐analytical choices, not interchangeable routes to background‐free CM (Pham et al. 2021; Shekari et al. 2023; Urzi et al. 2024).

Despite the experimental advantages of CM, isolating highly purified EV‐enriched fractions remains technically challenging. Traditional approaches, including ultracentrifugation, size‐exclusion chromatography, ultrafiltration, and immunoaffinity capture, involve trade‐offs in yield, purity, scalability, vesicle integrity, and carryover of non‐vesicular material or serum‐derived background (Mukerjee et al. 2025; Singh et al. 2024). Methodological primers and recent isolation reviews emphasize that no single separation method is universally optimal. Instead, method selection should be tailored to the specific downstream endpoint, acceptable co‐isolate burden, sample volume, and requirements for functional preservation (Aliakbari et al. 2024; Bharti et al. 2025). In practical terms, the enrichment method, acceptable recovery‐purity balance, and downstream endpoint require similar specifications, since the same preparation may not be equally suitable for omics profiling, functional assays, or translational product development (Welsh et al. 2024; Xu, Jin, et al. 2025).

For this reason, CM‐based EV studies are most informative when isolation is paired with transparent characterization and rigorous functional controls. At minimum, investigators should report culture format, cell density or confluence, viability, conditioning time, medium and serum‐depletion strategy, mycoplasma testing, CM processing, storage, freeze–thaw exposure, separation workflow, particle concentration and size distribution, morphology, EV‐associated markers, and likely non‐vesicular co‐isolates (Shekari et al. 2023; Welsh et al. 2024). In functional assays, EV‐depletion controls should ideally be complemented by add‐back or rescue experiments and, where mechanistically appropriate, by protease or RNase treatment, with and without membrane disruption, to distinguish contributions from soluble, surface‐associated, and luminal cargo (Welsh et al. 2024). When functional relevance is assigned to RNA cargo, these controls require parallel evidence for cargo protection or accessibility, uptake, and perturbation‐dependent functional effects (Mateescu et al. 2017; Welsh et al. 2024).

EV input should also be normalized and reported explicitly, for example by particle number, EV‐associated protein, donor‐cell number or cell‐equivalent input, and preferably accompanied by dose–response or time‐course assessment (Welsh et al. 2024). This aspect is especially important, given that different normalization strategies can change biological interpretation and may not be interchangeable across isolation methods or donor‐cell states. In translational or product‐development contexts, recent analyses of EV‐drug development similarly emphasize source definition, process control, dose metrics, potency readouts, release criteria, and batch consistency as prerequisites for reproducible EV preparations (Xu, Jin, et al. 2025). These metrics are complementary, as particle counts, total protein, lipid content, and donor‐cell‐equivalent input each capture different aspects of the preparation and may be influenced by non‐vesicular particles, soluble proteins, donor‐cell state, and recovery efficiency (Thery et al. 2018; Welsh et al. 2024).

Thus, refining CM workflows is essential not only for improving EV recovery, but also for strengthening causal interpretation. The major value of CM does not lie in eliminating variability; rather, it lies in explicitly defining, reporting, and experimentally controlling culture, isolation, characterization, and functional‐testing variables before findings are extended to biofluid‐derived EVs, animal models, or clinical settings. Such an experimental definition limits overinterpretation when CM‐derived findings are extrapolated to biofluid‐derived EVs, animal models, or clinical settings (Shekari et al. 2023; Welsh et al. 2024).

### 2.3 Size‐Based Isolation Techniques for CM‐Derived EVs

Size‐based EV isolation leverages differences in particle size and hydrodynamic behavior to enrich EV‐containing fractions from CM. Techniques such as ultrafiltration and size‐exclusion chromatography (SEC) use membrane filters or porous matrices to concentrate vesicles or separate them from larger cellular debris and, to some extent, from smaller soluble components (Shu et al. 2021; Zhang et al. 2020). These approaches are attractive for CM studies because they are relatively gentle, compatible with downstream molecular and functional analyses, and may better preserve vesicle integrity than harsh precipitation workflows (Zhang et al. 2020).

However, size‐based enrichment is not equivalent to analytical purity or subtype assignment. Protein aggregates, lipoproteins, extracellular ribonucleoprotein particles, serum‐derived particles, and other non‐vesicular components can overlap in size with EVs; therefore, these techniques generally yield EV‐enriched, rather than biogenetically or analytically pure, preparations (Shu et al. 2021; Welsh et al. 2024; Zhang et al. 2020). Consequently, downstream interpretation should regard the recovered material as an operationally defined EV‐enriched fraction unless cross‐validated characterization and functional controls support a stronger conclusion.

Recent literature supports a shift from single size‐based methods toward combined or hyphenated workflows. A recent review of modern EV isolation and separation techniques emphasizes that all isolation principles involve trade‐offs and that combined approaches can improve reliability by reducing interfering nanoscale contaminants (Liangsupree et al. 2026). In clinically oriented work, SEC separated EVs from 1 mL of plasma within 90 min and generated samples with suitable yield, purity, and a representative surface tetraspanin distribution, although this evidence derives from plasma rather than CM (Robinson et al. 2024).

For conditioned culture media, osmosis‐driven filtration has recently been used to concentrate EVs while removing smaller contaminants. The EV‐Osmoprocessor achieved a 50‐fold volume reduction in under 2 h, retained EVs, removed more than 99% albumin from cell‐conditioned culture medium, and improved particle‐to‐protein purity after subsequent SEC processing (Huang et al. 2025). Although this study used stem‐cell‐conditioned medium and not tumor‐cell CM, it supports the broader principle that concentration‐plus‐SEC workflows can improve analytical performance when matrix‐specific recovery and purity metrics are reported.

Analytical SEC developments also emphasize the need to couple size‐based isolation with multiparametric characterization. Recent SEC‐based characterization workflows combine particle, protein, fluorescence, immunolabeling, and light‐scattering readouts to monitor both EV‐associated particles and smaller impurities, making them useful for method development and quality control but not for proving EV subtype identity (Bozic et al. 2025).

In CM‐derived tumor EV studies, the most defensible use of size‐based isolation is therefore enrichment within a fit‐for‐purpose workflow. Reports should specify filter or membrane cut‐off, SEC column and fraction‐selection criteria, input volume, recovery, particle‐to‐protein ratio or equivalent purity metric, EV‐associated marker profile, co‐isolate assessment, storage conditions, and how the preparation is normalized for functional assays. This level of reporting is essential since method‐dependent differences in recovery and co‐isolation can alter cargo profiles and apparent biological activity.

### 2.4 Ultracentrifugation

Ultracentrifugation, particularly differential ultracentrifugation (dUC), remains one of the most widely used size‐ and sedimentation‐based approaches for enriching EV‐containing fractions from CM and other biological matrices (Doyle and Wang 2019). A typical workflow uses sequential low‐ and intermediate‐speed centrifugation steps to remove cells, apoptotic bodies, and larger extracellular particles, followed by a high‐speed step, often around 100,000 × *g*, to pellet small EV‐enriched fractions; the pellet is then washed and resuspended for downstream analyses (Zhang et al. 2020). Considering that sedimentation depends not only on nominal relative centrifugal force but also on rotor geometry, *k*‐factor, run time, temperature, sample viscosity, and fill volume, protocols that report only an approximate *g*‐force are difficult to compare directly across laboratories (Ljungstrom and Oltra 2025; Welsh et al. 2024).

For CM‐derived tumor EV studies, dUC is attractive as it can process relatively large volumes without introducing polymeric precipitation reagents, although it remains an enrichment strategy, not a purification or subtype‐defining procedure. Repeated high‐speed centrifugation can reduce recovery, promote vesicle aggregation or distortion, and co‐pellet protein aggregates, ribonucleoprotein complexes, serum‐derived particles, and other non‐vesicular material that may overlap with EVs in sedimentation behavior (Ljungstrom and Oltra 2025; Lobb et al. 2015; Zhang et al. 2020). These limitations are especially relevant when CM is generated in EV‐depleted serum‐containing media, because residual serum‐derived components and soluble tumor‐cell products may be recovered together with the EV‐enriched pellet and can influence proteomic, RNA, or functional readouts (Welsh et al. 2024).

Density‐cushion or density‐gradient ultracentrifugation can improve separation by providing buoyant‐density information, but these approaches increase processing time and technical complexity and, by themselves, do not establish exosomal biogenesis or analytical purity. Current reporting recommendations therefore emphasize transparent documentation of pre‐processing conditions, rotor type, centrifugal force, time, temperature, wash steps, sample volume, and post‐isolation characterization, together with independent complementary readouts of particle number, morphology, EV‐associated markers, and non‐vesicular co‐isolates (Welsh et al. 2024). Recent methodological reviews also support the view that combined or hyphenated workflows, for example, dUC followed by SEC or other polishing steps, are often better suited than a single isolation principle when purity and functional attribution are priorities (Liangsupree et al. 2026).

Accordingly, ultracentrifugation remains a useful reference and discovery‐stage method for CM‐derived EV research. However, it does not constitute a universally optimal, clinically scalable, or purity‐defining standard. For mechanistic studies, dUC‐derived preparations should be explicitly regarded as EV‐enriched fractions and interpreted only after complementary characterization, dose normalization, and functional controls that address co‐isolated soluble or particulate material (Aliakbari et al. 2024; Welsh et al. 2024).

### 2.5 Ultracentrifugation Versus Ultrafiltration

Comparisons between ultracentrifugation and ultrafiltration should highlight method‐specific trade‐offs, not suggest that one method is universally superior. In the study by Lobb et al., ultrafiltration recovered more particles below 100 nm from conditioned medium and substantially reduced processing time, requiring approximately 20 min for 150 mL of medium compared with approximately 180 min for ultracentrifugation (Lobb et al. 2015). This finding supports the practical value of ultrafiltration for rapidly concentrating CM‐derived EV‐enriched fractions, but it does not establish greater purity, subtype identity, or functional equivalence across downstream applications.

Ultracentrifugation remains useful when relatively large CM volumes must be processed without introducing membrane materials, but repeated high‐speed centrifugation can reduce recovery, promote aggregation or vesicle deformation, and co‐pellet protein aggregates or other non‐vesicular material (Lobb et al. 2015; Zhang et al. 2020). Ultrafiltration can be faster, less equipment‐intensive, and easier to scale for parallel sample handling; however, its performance depends on membrane material, molecular‐weight cutoff or pore characteristics, sample viscosity, pre‐clearing steps, centrifugal force or pressure, washing strategy, and risk of membrane fouling, EV adsorption, or concentration of soluble co‐isolates (Ansari et al. 2024; Chaturvedi et al. 2026). Thus, ultrafiltration appears to be a better concentration and enrichment strategy, not a stand‐alone guarantee of analytical purity.

Lobb et al. also reported broadly comparable particle yields and slightly higher detection of TSG101 in ultrafiltration‐derived preparations (Lobb et al. 2015). However, TSG101 abundance alone does not establish purity or subtype identity, given that EV preparations require cross‐validated characterization of particle number and size, morphology, EV‐associated markers, and non‐vesicular co‐isolates (Welsh et al. 2024). This point is especially important for CM studies, where residual serum‐derived particles, soluble proteins, ribonucleoprotein complexes, and protein aggregates may be retained or concentrated differently by ultracentrifugation and ultrafiltration.

Therefore, instead of a single preferred isolation method, recent methodological literature favors fit‐for‐purpose workflows. Modern reviews emphasize that combined or hyphenated approaches are often needed to balance recovery, purity, scalability, and compatibility with downstream omics or functional assays (Liangsupree et al. 2026). Filter‐based strategies are also evolving beyond simple volume reduction. For example, filter‐aided EV enrichment approaches such as FAEVEr use molecular‐weight cutoff filtration and washing to retain EV particles while reducing interfering non‐EV proteins for proteomic workflows (Pauwels, Van de Steene, De Muyer, et al. 2025; Pauwels, Van de Steene, Van de Velde, et al. 2025). These developments support the use of ultrafiltration as part of optimized CM‐EV workflows, particularly when followed by SEC, density‐gradient separation, immunoaffinity enrichment, or rigorous analytical validation.

Accordingly, the choice between ultracentrifugation and ultrafiltration should be guided by the intended endpoint. Proteomic profiling may prioritize removal of abundant soluble proteins; RNA or miRNA studies may prioritize recovery and nuclease‐free handling; functional assays require additional controls for dose normalization, co‐isolates, and EV‐specific causality. In all cases, method reporting must include sample volume, pre‐clearing steps, rotor or filter specifications, centrifugal force or pressure, wash conditions, recovery estimates, and characterization readouts, in line with current EV guidance (Welsh et al. 2024). A comparative summary is provided in Table 1.

**TABLE 1:** Comparative analysis of ultracentrifugation and ultrafiltration for EV enrichment from CM.

Parameter	Ultracentrifugation	Ultrafiltration	Interpretive note
Processing time for 150 mL CM	Approximately 180 min in the Lobb et al. comparison	Approximately 20 min in the Lobb et al. comparison	Speed advantage does not establish higher purity or functional equivalence.
Particle recovery	Moderate recovery of small particles in the cited comparison	Higher recovery of particles below 100 nm in the cited comparison	Recovery should be interpreted in the context of size distribution, particle/protein ratio, and cross‐validated markers.
EV‐associated marker detection	Baseline TSG101 detection in the cited comparison	Slightly higher TSG101 detection in the cited comparison	TSG101 supports EV enrichment but is not a stand‐alone marker of purity or subtype.
Major operational strengths	Can process large volumes; no membrane material introduced; widely used reference method.	Fast, accessible, scalable for parallel processing, useful for concentration before further purification	The best method depends on the downstream endpoint and the acceptable co‐isolate burden.
Major limitations	Time‐consuming; specialized equipment; aggregation, deformation, EV loss, and co‐pelleting risk	Membrane clogging/fouling; EV adsorption; filter‐to‐filter variability; concentration of soluble contaminants	Both methods yield operational EV‐enriched fractions rather than analytically pure or biogenesis‐defined exosomes.
Best‐practice use	Useful when paired with washing, density‐based refinement, or independent complementary characterization	Useful as a concentration/enrichment step, often before SEC, affinity capture, or omics analysis	Report method parameters in detail, including characterization and functional controls.

*Note:* Processing time, particle recovery, and TSG101 detection are derived from the conditioned‐medium comparison by (Lobb et al. 2015). General interpretive limitations, including co‐isolation, operational EV‐enriched status, subtype and purity constraints, and reporting expectations, should be evaluated in light of EV characterization and reporting guidance from (Welsh et al. 2024) and recent reviews of EV isolation methods by (Liangsupree et al. 2026).

### 2.6 Size Exclusion Chromatography

Size‐exclusion chromatography (SEC) is a widely used size‐based approach for enriching EV‐containing fractions from conditioned medium (CM). It separates vesicles and soluble macromolecular components based on differences in hydrodynamic size, regardless of vesicle biogenesis. After CM pre‐clearing and concentration, often by ultrafiltration, the sample is loaded onto a pre‐equilibrated SEC column. Larger particles are excluded from the porous matrix and elute in earlier fractions, whereas smaller soluble proteins and other low‐molecular‐weight components enter the pores and elute later. This aspect permits the collection of EV‐enriched fractions with reduced soluble‐protein carryover (Shu et al. 2021).

SEC has several methodological strengths in CM‐derived EV studies. By avoiding high centrifugal forces and polymer‐based precipitation, SEC is relatively gentle, helping preserve vesicle integrity while reducing soluble‐protein contamination. SEC‐compatible fractions are therefore suitable for downstream proteomic, transcriptomic, and functional analyses, provided that particle recovery, fraction selection, and sample normalization are reported transparently (Shu et al. 2021).

Nevertheless, the SEC should be regarded as an enrichment or polishing strategy, not as a stand‐alone purification or subtype‐defining method. It does not, by itself, generate analytically pure EV preparations or biogenetically defined exosome fractions, and its performance depends on the column matrix and bed volume, sample input and viscosity, matrix complexity, fraction‐collection criteria, and the extent to which SEC is combined with upstream concentration or downstream characterization. For CM‐derived tumor EV studies, reports require specification of pre‐clearing and concentration steps, column type, loaded volume, collected fractions, recovery and particle‐to‐protein metrics where available, EV‐associated markers, and assessment of likely soluble or particulate co‐isolates, so that downstream functional effects can be attributed with appropriate caution (Welsh et al. 2024). Recent comparative work in plasma‐derived EVs shows that SEC column choice can markedly affect EV recovery, separation efficacy, relative purity, and miRNA‐cargo analysis (Bracht et al. 2024). Although this evidence is not CM‐specific, it reinforces the need to report SEC configurations in detail and to avoid treating them as interchangeable when SEC is applied to CM‐derived EV‐enriched fractions (Bracht et al. 2024). Multiparametric SEC workflows further support coupling fractionation with particle, protein, fluorescence, immunolabeling, and light‐scattering readouts to distinguish EV‐associated fractions from smaller impurities and free labeling components (Bozic et al. 2025).

### 2.7 Immunoaffinity‐Based Isolation Techniques

Immunoaffinity‐based methods rely on antibodies directed against accessible surface antigens associated with selected EV populations. These antibodies can be immobilized on magnetic beads, plates, resins, or microfluidic devices to enrich subsets of EVs carrying the target epitope (Doyle and Wang 2019). In CM‐derived tumor EV studies, this selectivity is useful when the experimental question concerns a marker‐defined EV subset. However, it also means that antigen‐negative vesicles are systematically under‐represented (Welsh et al. 2024). For example, ASGR1 has been used to enrich hepatocyte‐derived EVs (Conde‐Vancells et al. 2008). This example illustrates lineage‐marker‐based enrichment and is not generalizable to tumor‐derived EV capture unless the selected antigen is biologically justified for the donor‐cell context. A key limitation of this strategy is that it depends on the availability and accessibility of appropriate surface epitopes; internal vesicle cargo cannot be targeted without membrane disruption, which limits the approach to surface‐accessible features (Li et al. 2017). Capture efficiency is also affected by antigen density, epitope masking, antibody clone, immobilization chemistry, incubation time, washing stringency, and elution conditions (Fernandes et al. 2025; Welsh et al. 2024). Recent immuno‐affinity chromatography work using clarified cell‐culture feedstocks further supports phenotype‐guided marker selection and process‐parameter reporting for affinity‐based EV capture workflows (Fernandes et al. 2025). In addition, although immunoaffinity approaches often yield lower total recovery, they can provide greater enrichment of selected surface‐marker‐defined EV subpopulations than non‐selective bulk isolation methods (Fernandes et al. 2025; Li et al. 2017; Welsh et al. 2024). Accordingly, immunoaffinity capture represents selective enrichment of surface‐marker‐defined EV subsets, not recovery of the total CM‐derived EV population or proof of exosomal biogenesis (Welsh et al. 2024).

**FIGURE 2:**
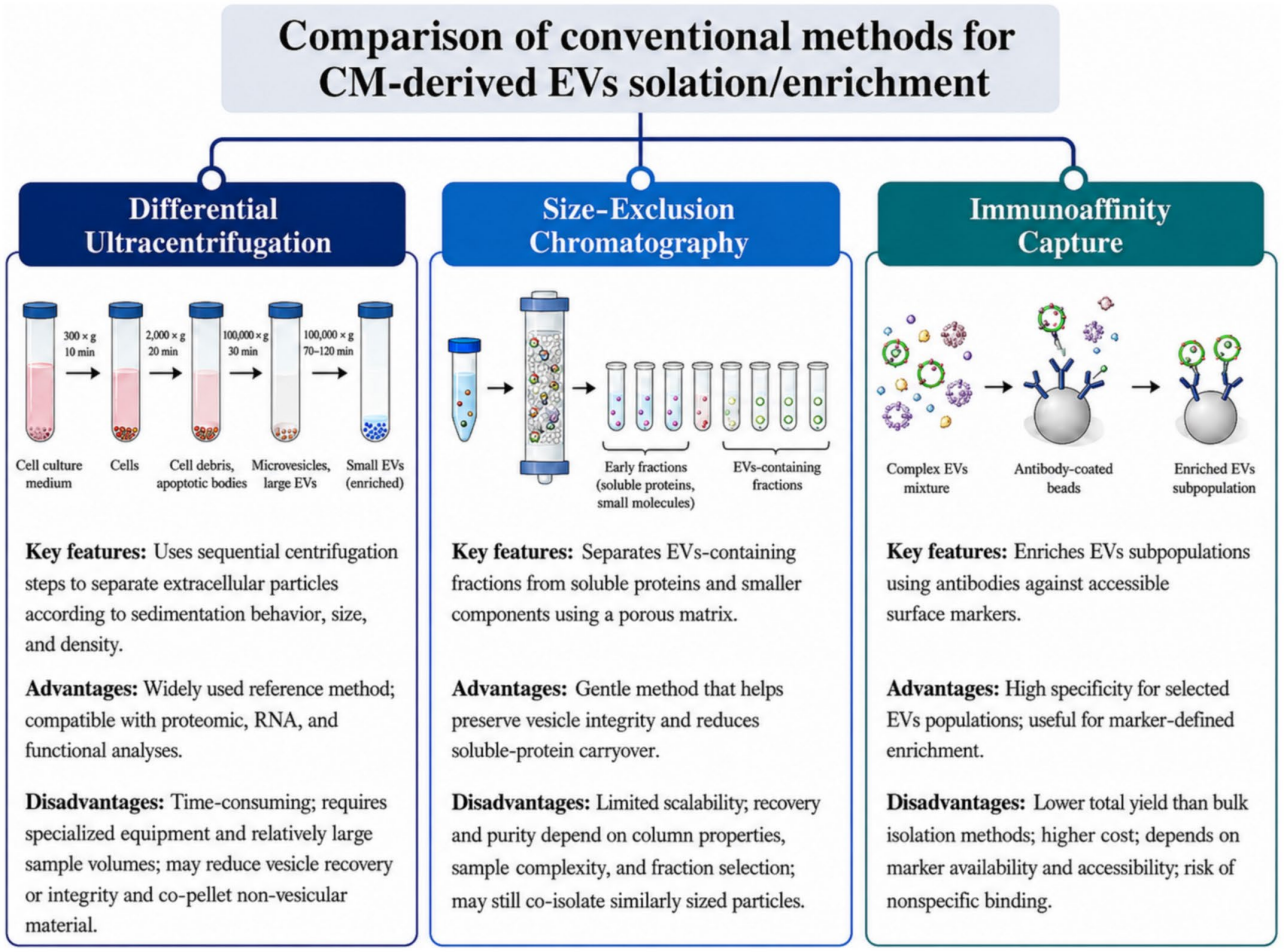
Overview of conventional methods for CM‐derived EV isolation and enrichment. Differential ultracentrifugation uses sequential centrifugation steps to separate extracellular particles based on sedimentation behavior, size, and density. However, it is time‐consuming, requires specialized equipment and relatively large sample volumes, and may reduce vesicle recovery or integrity due to co‐pelleting, aggregation, or mechanical stress. Size‐exclusion chromatography separates EV‐containing fractions from soluble proteins and smaller components using a porous matrix, providing relatively gentle processing and reduced soluble‐protein carryover, although recovery and purity depend on column properties, sample complexity, and fraction selection. Immunoaffinity capture enriches surface‐marker‐defined EV subpopulations by targeting accessible surface antigens with antibodies, thereby increasing specificity for the targeted phenotype but typically reducing total yield and increasing cost, while excluding antigen‐negative EVs from the captured fraction. It also requires careful controls for antibody specificity, epitope accessibility, and possible co‐capture of non‐vesicular antigen‐bearing material. The choice of method should be guided by the sample source, downstream application, scalability requirements, and acceptable trade‐offs among recovery, specificity, and co‐isolation of non‐vesicular material. No single workflow should be interpreted as proof of EV purity or exosomal origin without complementary and methodologically independent characterization. Therefore, the immunoaffinity component of this figure represents selective marker‐defined enrichment or detection, not proof of total EV recovery, purity, or exosomal origin.

A CM‐specific example of this approach was reported by Sundaram et al., who developed a multilayer micro‐nanofluidic device for isolating EV‐enriched fractions from liposarcoma cell‐conditioned medium. Their platform combined tangential flow separation with immunoaffinity capture, thereby integrating size‐based sorting and antigen‐based retrieval within a single system. In that study, the reported EV recovery rate was approximately 84% on that platform, markedly higher than that obtained with ultracentrifugation (~6%) or ExoQuick (~16%) (Sundaram et al. 2020). These recovery values should be regarded as platform‐specific, not as generalizable performance benchmarks, because recovery depends on sample preprocessing, marker expression, device architecture, washing and elution conditions, and the analytical method used to quantify recovered EVs. More recent breast cancer cell culture work using a CD63/CD81‐directed nanostructured microfluidic chip further illustrates that antibody‐based platforms can capture marker‐positive EVs directly from CM (Cabeza et al. 2025). This finding supports targeted detection or enrichment of selected marker‐positive EV fractions (Welsh et al. 2024). Figure 2 provides a comparative overview of conventional EV isolation methods, including differential ultracentrifugation, size‐exclusion chromatography, and immunoaffinity capture, highlighting their principal advantages, limitations, and representative applications within a fit‐for‐purpose CM‐EV workflow.

For CM‐derived tumor EV studies, immunoaffinity workflows should therefore report the target antigen and selection rationale, antibody clone and immobilization format, input volume and pre‐clearing steps, incubation and wash conditions, elution procedure, recovery and purity metrics, negative and isotype controls, and downstream EV characterization (Fernandes et al. 2025; Welsh et al. 2024). Where the captured material is used in functional assays, dose normalization and controls for soluble or antigen‐bearing non‐vesicular co‐isolates remain necessary. This level of reporting is important because immunocapture can enrich biologically informative EV subpopulations while also introducing selection bias that affects cargo profiling and functional interpretation (Welsh et al. 2024).

### 2.8 Characterization, Purity, and Reproducibility of CM‐Derived EV Preparations

Although CM provides a controlled source of EVs from defined donor cells, it is not a standardized or contaminant‐free matrix; CM‐derived preparations are not intrinsically standardized, because EV yield, composition, co‐isolate burden, and apparent bioactivity can vary according to serum depletion strategy, cell density and confluence, culture configuration, conditioning interval, mycoplasma status, and downstream handling of the conditioned medium, including variables that affect both analytical readouts and downstream functional interpretation (Shekari et al. 2023; Welsh et al. 2024). Isolation alone therefore does not establish EV identity or purity, nor does it define vesicle subtype, cellular origin, or freedom from co‐isolated material (Welsh et al. 2024).

At a minimum, CM‐derived preparations require complementary characterization approaches that address particle abundance and size distribution, morphology, EV‐associated markers, and the likely presence of non‐vesicular co‐isolates and serum‐derived background, using a multiparametric strategy (Lai et al. 2022; Zhang et al. 2020). This requirement is consistent with EV reporting guidance, which represents a minimal reporting framework (Welsh et al. 2024). Morphological assessment by electron microscopy and particle sizing by nanoparticle tracking analysis or related methods may support EV characterization, but these readouts require combined interpretation and cannot be used as stand‐alone proof of vesicle identity, purity, or subtype (Lai et al. 2022; Zhang et al. 2020).

Likewise, markers such as CD63, CD81, TSG101, and ALIX can support the characterization of EV‐enriched fractions, but none alone establishes purity, subtype identity, or endosomal origin, even when these markers are detected together with canonical tetraspanins or ESCRT‐associated proteins (Welsh et al. 2024). Recent single‐particle nanoflow cytometry work further supports this multiparametric characterization logic by showing that high‐resolution analysis can measure EV‐associated size, concentration, and tetraspanin expression while also revealing exogenous particle contamination from culture media supplements (Vyzourek et al. 2025). Automated cryo‐EM combined with supervised machine learning has likewise been reported to improve reproducible visualization and classification of EVs versus co‐isolating lipoproteins or protein aggregates, reinforcing that purity assessment benefits from complementary structural and single‐particle methods (Enciso‐Martinez et al. 2026).

Importantly, purity in EV research is generally relative, and preparations enriched in small EVs may still contain soluble proteins, lipoprotein‐like particles, serum‐derived EVs, or other extracellular components, including extracellular ribonucleoprotein particles and protein aggregates (Zhang et al. 2020). This limitation is consistent with EV reporting guidance and underscores the need to report separation performance, recovery, and co‐isolate assessment (Welsh et al. 2024). Transparent reporting of culture conditions, CM handling, isolation workflow, and analytical readouts is therefore essential for reproducibility and for meaningful cross‐study comparison, particularly when EV preparations are compared across cell lines, culture formats, treatments, or laboratories (Shekari et al. 2023; Welsh et al. 2024). Accordingly, statements about reproducibility should specify whether they refer to EV recovery, EV‐associated molecular readouts, co‐isolate reduction, or functional bioactivity, because these endpoints are not interchangeable and may respond differently to culture and separation variables (Shekari et al. 2023; Welsh et al. 2024).

### 2.9 Functional Causality and EV Dose Normalization in CM‐Based Studies

CM‐based workflows are particularly useful for mechanistic studies because they allow investigators to recover EV‐enriched fractions from relatively large volumes of conditioned supernatant, thereby obtaining sufficient material for in vitro functional assays and, in some settings, in vivo administration (Singh et al. 2024; Zhang et al. 2020). They also support comparative experimental designs, for example, by isolating EVs from different cancer cell lines or from the same donor cells under distinct treatment or microenvironmental conditions, to examine how EV cargo and function vary with tumor type or experimental perturbation (Dowling and Clynes 2011; Singh et al. 2024). Because culture conditions and pre‐analytical variables can affect the release, biochemical composition, and functional properties of cell culture‐conditioned medium‐derived EVs, this design is most informative when whole‐CM activity, EV‐enriched fraction activity, and recipient‐cell response are treated as experimentally distinct readouts of the same biological process (Shekari et al. 2023; Welsh et al. 2024).

However, CM‐based experiments strengthen mechanistic inference only when EV‐specific causality is tested explicitly (Welsh et al. 2024). EV‐depleted CM controls are informative, but causal inference is stronger when complemented by add‐back or rescue designs, multiparametric controls addressing soluble co‐isolates, and, where mechanistically appropriate, protease or RNase treatment with and without membrane disruption to distinguish surface‐associated, luminal, and freely soluble cargo (Welsh et al. 2024). For EV‐associated RNA‐cargo claims, such controls are particularly important because functional attribution requires evidence for cargo accessibility, uptake, and perturbation‐dependent effects (Mateescu et al. 2017). EV input also requires clear normalization and reporting, for example by particle number, EV‐associated protein, or cell‐equivalent donor input, with incorporation of dose–response or time‐course designs whenever feasible (Welsh et al. 2024). Because particle number, total EV‐associated protein, lipid content, and donor‐cell‐equivalent input capture different properties of the preparation, the selected dose metric needs justification, transparent reporting, and, where feasible, a second normalization metric or dose–response testing (Thery et al. 2018; Welsh et al. 2024). Without this level of experimental definition, apparent EV effects may reflect differences in preparation load, co‐isolate content, or assay context, not bona fide EV‐mediated signaling (Welsh et al. 2024). A stronger causal design therefore compares whole CM, EV‐enriched fractions, EV‐depleted CM, and add‐back of the isolated EV fraction, because EV depletion alone may not exclude residual or redistributed contributions from soluble mediators, extracellular ribonucleoprotein complexes, protein aggregates, and other co‐isolated or culture‐derived material (Mateescu et al. 2017; Shekari et al. 2023; Welsh et al. 2024). Enzymatic controls require similarly careful interpretation: RNase or protease treatment in the absence of detergent primarily interrogates exposed or co‐isolated cargo, whereas detergent‐permeabilized conditions can help assess luminal cargo contributions only if vesicle disruption and cargo accessibility are experimentally verified (Mateescu et al. 2017; Welsh et al. 2024). This distinction is particularly important in CM‐derived tumor EV studies, where cytokines, metabolites, extracellular ribonucleoprotein complexes, protein aggregates, and residual serum‐derived material may modify recipient‐cell phenotypes independently of vesicle‐mediated cargo transfer (Shekari et al. 2023; Welsh et al. 2024).

**FIGURE 3:**
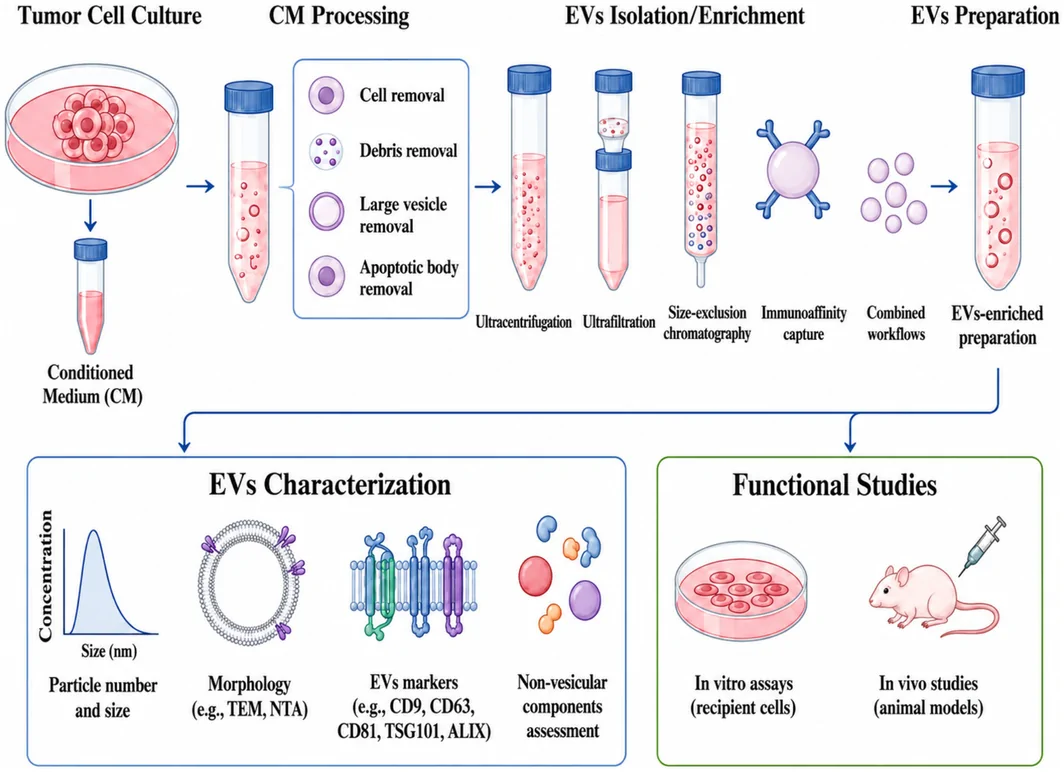
CM‐based workflow for EV isolation, characterization, and downstream functional testing. Tumor cells cultured under defined conditions release extracellular vesicles (EVs) and soluble factors into conditioned medium (CM). After CM collection, sequential processing steps remove cells, debris, apoptotic bodies, and larger vesicles before EV‐containing fractions are enriched by ultracentrifugation, ultrafiltration, size‐exclusion chromatography, immunoaffinity capture, or a combination of these methods. The resulting EV‐enriched preparation is characterized using complementary approaches that assess particle number and size distribution, morphology, EV‐associated markers, and non‐vesicular components. These preparations can then be evaluated in functional studies, including recipient‐cell assays in vitro and animal models in vivo. Appropriate experimental controls include, whenever feasible, comparisons of whole CM, EV‐depleted CM, EV‐enriched fractions, and EV‐add‐back or rescue conditions; enzymatic controls with and without membrane disruption when mechanistically appropriate; dose normalization; and controls for soluble or particulate co‐isolates. These controls strengthen causal interpretation by distinguishing EV‐associated biological effects from preparation load, co‐isolate burden, and assay‐context effects.

Taken together, these considerations explain why CM remains a powerful platform for EV research: not because it eliminates variability, but because it enables variability to be defined, reported, and experimentally controlled when culture conditions, isolation methods, characterization, dosing, and functional controls are integrated into the experimental design (Shekari et al. 2023; Welsh et al. 2024). Figure 3 summarizes a representative conditioned medium (CM)‐based workflow for EV isolation and characterization, and potentially applicable downstream functional testing (Welsh et al. 2024).

## 3 Mechanistic Insights From CM Studies

CM‐based studies have been central for dissecting how tumor‐derived EVs, including small EV‐enriched preparations frequently reported as exosomes in the primary literature, contribute to cancer progression through defined donor‐recipient cell interactions. Here, the term exosome is retained only when it reflects the terminology or biogenetic interpretation of the cited study; otherwise, tumor‐derived EVs or EV‐enriched preparations are used as operational descriptors, consistent with current EV nomenclature guidance (Welsh et al. 2024). This distinction is important since CM workflows often enrich heterogeneous vesicle populations and may also carry soluble or non‐vesicular co‐isolates, so mechanistic attribution requires careful linkage to the EV fraction and to the experimental controls used (Shekari et al. 2023; Welsh et al. 2024).

Recent syntheses of cancer EV biology reinforce that tumor‐derived EVs can influence multiple cancer‐associated programs, including pre‐metastatic niche formation, angiogenesis, immune evasion, stromal reprogramming, metabolic adaptation, and therapeutic resistance (Semeradtova et al. 2025). These effects are commonly attributed to the transfer or presentation of proteins, lipids, DNA, mRNAs, non‐coding RNAs, and other EV‐associated cargos to endothelial, fibroblast, immune, stromal, and malignant recipient cells (Semeradtova et al. 2025). However, the functional outcome is not uniform: it depends on tumor type, donor‐cell state, culture configuration, microenvironmental stressors such as hypoxia or treatment exposure, EV‐isolation strategy, recipient‐cell identity, and the contribution of soluble or particulate co‐isolates (Shekari et al. 2023; Welsh et al. 2024).

Accordingly, the following subsections emphasize mechanistic insights that can be most reliably attributed to controlled in vitro, ex vivo, and selected in vivo systems utilizing CM‐derived or tumor‐derived EV‐enriched preparations. The discussion is structured around four principal functional axes: metastasis and pre‐metastatic niche formation; immunosuppression and immune evasion; stromal reprogramming and angiogenesis; and therapeutic resistance. Across these axes, the most robust causal inferences are drawn from studies employing EV enrichment with EV‐depleted controls, add‐back or rescue experiments, cargo perturbation, uptake or release blockade, dose normalization, and rigorous controls for soluble mediators and co‐isolated extracellular material (Welsh et al. 2024).

### 3.1 Metastasis and Pre‐Metastatic Niche Formation

**FIGURE 4:**
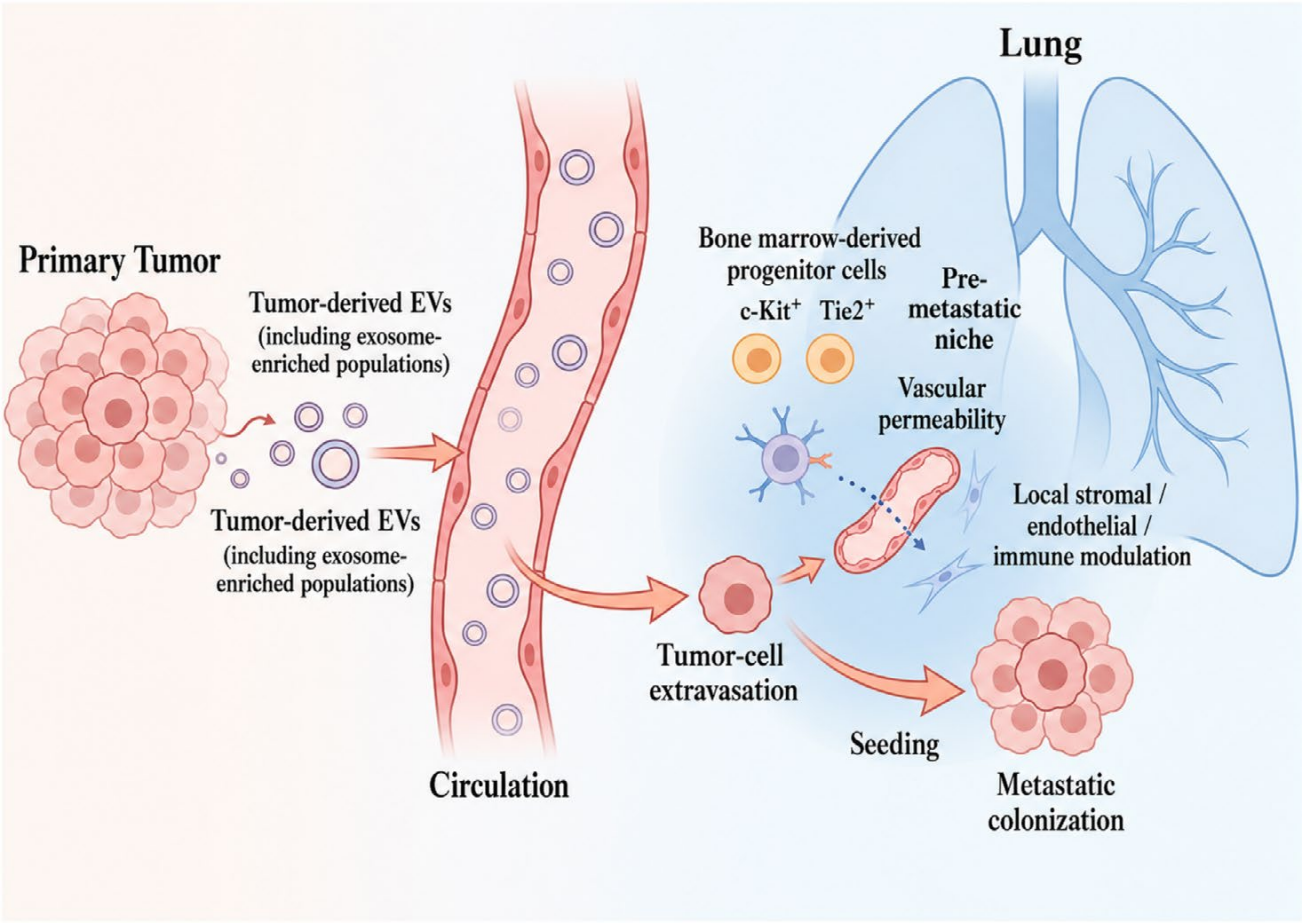
Tumor‐derived EVs contribute to pre‐metastatic niche formation and metastatic colonization. Primary tumor cells release extracellular vesicles (EVs), including small EV‐enriched fractions commonly referred to as exosomes, into the circulation. Tumor‐derived EVs can reach distant organs such as the lung, where they promote the formation of a pre‐metastatic niche by increasing vascular permeability and modulating local stromal, endothelial, and immune cell populations. Furthermore, these EVs can influence bone marrow‐derived progenitor cells, including c‐Kit+ and Tie2+ populations, thereby further supporting niche development. Collectively, these alterations generate a microenvironment that facilitates tumor cell extravasation, seeding, and metastatic colonization. The figure presents a synthesis of model‐based evidence and does not suggest a uniform pathway for all tumor types.

One of the clearest mechanistic contributions of CM‐based studies is evidence that tumor‐derived EV‐enriched preparations can prime distant tissues before the arrival of overt cancer cells, a process commonly described as pre‐metastatic niche formation (Peinado et al. 2012). In the seminal melanoma study by Peinado et al., exosome‐enriched conditioned medium from highly metastatic cells was used in bone marrow and in vivo models, showing that these preparations can educate bone marrow progenitor cells and stromal components toward a metastasis‐supporting state (Peinado et al. 2012). Mechanistically, MET receptor tyrosine kinase transfer was associated with a pro‐vasculogenic phenotype, including c‐Kit+ and Tie2+ progenitor populations, and with vascular leakiness at future metastatic sites, such as the lung (Peinado et al. 2012). Depletion of MET in the vesicle preparation reduced bone marrow reprogramming and metastatic promotion, supporting a cargo‐dependent, but model‐specific, causal role (Peinado et al. 2012). Figure 4 summarizes how tumor‐derived EVs can contribute to pre‐metastatic niche formation by modulating bone marrow‐derived cells, vascular permeability, and immune or stromal components at distant sites.

This interpretation should remain operational, as a recent review of in vivo tumor‐derived EV studies in pre‐metastatic and metastatic niches found substantial methodological heterogeneity and noted that many models rely on exogenous administration of EVs prepared in vitro, in preference to endogenous tracking of tumor‐released EVs (Blavier et al. 2026).

CM and in vivo studies have also implicated EV‐associated integrins as determinants of organotropic metastasis. Hoshino et al. showed that exosome‐enriched preparations from different tumor types displayed distinct integrin patterns and were preferentially taken up by specific resident organ cells (Hoshino et al. 2015). In that study, vesicles from lung‐tropic breast cancer models preferentially interacted with lung fibroblasts and epithelial cells, whereas vesicles from liver‐tropic models targeted Kupffer cells, thereby conditioning organ‐specific pre‐metastatic niches (Hoshino et al. 2015). Proteomic analyses identified integrin alpha6beta4 and alpha6beta1 as enriched in lung‐tropic vesicles and integrin alphavbeta5 as enriched in liver‐tropic vesicles (Hoshino et al. 2015). Functional blockade of these integrins reduced vesicle uptake by target organ cells and decreased organ‐specific metastasis, supporting a causal role for EV‐associated integrins in that experimental context (Hoshino et al. 2015).

Recent work has further broadened the immune‐remodeling dimension of pre‐metastatic niche formation. In osteosarcoma models, tumor cell‐derived EVs carrying S100A11 activated lung interstitial macrophages and promoted CXCL2‐dependent recruitment of granulocytic myeloid‐derived suppressor cells, thereby linking a defined EV protein cargo to immunosuppressive lung niche formation and metastatic colonization (Deng et al. 2024). In colorectal cancer liver metastasis models, circ‐0034880‐enriched tumor EVs were reported to enter liver tissue and activate SPP1highCD206+ pro‐tumor macrophages; pharmacological targeting with ginsenoside Rb1 reduced this EV‐associated macrophage program and impaired pre‐metastatic niche formation in that experimental system (Zhou, Song, et al. 2024). These examples strengthen the concept that tumor‐derived EVs can shape metastatic organ competence through macrophage and myeloid‐cell reprogramming, while also reinforcing the need to specify tumor type, target organ, EV cargo, and experimental model before attributing direct causal effects.

Additional studies expand the organotypic niche‐priming framework to include metabolic remodeling. Exosome‐enriched preparations from cisplatin‐induced dormant lung cancer cells carried IGF‐2 and IGFBP2 and, after uptake by bone marrow stromal cells (BMSCs), activated IGF‐1R signaling and shifted BMSCs toward glycolysis. This metabolic reprogramming supported metastatic seeding and growth through a mechanism consistent with the reverse Warburg effect (Xu et al. 2022). Inhibition of lactate production or IGF‐1R signaling attenuated A549 cell metastasis, strengthening the functional link between vesicle‐associated metabolic crosstalk and niche formation in this model.

Similarly, EV‐associated ADAM17 derived from colorectal cancer (CRC) cells has been implicated in increased vascular permeability and hematogenous dissemination. Specifically, ADAM17‐positive tumor‐derived exosomes were shown to alter VE‐cadherin membrane localization, increase endothelial barrier leakiness, promote pre‐metastatic niche formation, and facilitate CRC extravasation in vivo (Li, Xue, et al. 2024). Evidence from endothelial permeability assays and mouse models supports these observations. However, ADAM17 should be considered a context‐specific candidate biomarker and therapeutic target, rather than a universal marker of extracellular vesicle (EV)‐mediated metastasis across all tumor types.

Additional vascular‐ and hypoxia‐regulated EV programs further support the context‐dependent nature of niche priming. In gastric cancer, CD147‐high EVs were reported to activate VEGF/AKT/eNOS/NO and AKT/mTOR/p70S6K signaling, disrupt endothelial barrier integrity, increase vascular leakage, and promote metastatic tumor formation (Zhang, Zhao, et al. 2025). In non‐small cell lung cancer, hypoxia‐induced exosomal circPLEKHM1 promoted macrophage polarization toward a metastasis‐supporting phenotype through PABPC1‐eIF4G‐dependent regulation of oncostatin M receptor translation, and circPLEKHM1‐targeted intervention reduced metastasis in vivo in that model (Wang et al. 2024a). These findings link vascular permeability, hypoxia‐induced EV cargo, macrophage education, and metastatic progression, but they are not generalizable across tumor types without model‐specific validation.

EV‐mediated niche formation can also involve resident immune cells. In brain metastasis models of lung cancer, tumor‐derived exosomes taken up by brain endothelial cells were linked to Dickkopf‐1 (Dkk‐1)‐dependent changes that favored microglial acquisition of a pro‐tumorigenic phenotype, thereby contributing to a permissive neurovascular niche (Gan et al. 2020). Given that this pathway involves sequential interactions among tumor cells, endothelial cells, and microglia, this mechanism is not universal to brain metastasis.

Emerging data also broaden the niche concept beyond tumor‐cell‐derived EVs alone. In a 2025 Cell study, small EVs released from CXCL13‐reprogrammed interstitial macrophages in the non‐metastatic lung microenvironment carried clustered integrin beta2 and promoted platelet aggregation, cancer‐associated thrombosis, and lung metastasis; blockade of integrin beta2 decreased both sEV‐induced thrombosis and lung metastasis (Lucotti et al. 2025). These observations provide indirect evidence for CM‐derived tumor EVs and are relevant because they show that systemic cancer progression can generate host‐derived EV niches that amplify metastatic competence.

Collectively, these findings indicate that tumor‐derived EVs can contribute to organ‐specific pre‐metastatic niche formation through metabolic reprogramming, vascular destabilization, immune modulation, and stromal cell education. Strong mechanistic support comes from studies that combine CM‐ or tumor‐derived EV preparations with in vivo tracing, cargo perturbation, uptake blockade, or add‐back/rescue strategies. These mechanisms remain attractive translational targets; however, their diagnostic and therapeutic value requires tumor‐type‐specific validation. Therefore, accurate interpretation of pre‐metastatic niche mechanisms requires distinguishing between exogenous extracellular vesicle (EV) administration models and endogenous EV‐tracking approaches. It is essential to avoid extrapolating conditioned medium (CM)‐derived or tumor‐derived EV effects to clinical interventions without validating the EV source, dose, biodistribution, cargo dependence, and co‐isolate controls (Blavier et al. 2026; Welsh et al. 2024).

### 3.2 Immunosuppression and Immune Evasion

Tumor‐derived EVs can also contribute to immune escape by transferring immunoregulatory cargo to immune cells. A prominent example is PD‐L1 carried by tumor‐derived EVs or exosome‐enriched preparations. Using vesicles purified from lung cancer cell culture media, Kim et al. showed that PD‐L1‐positive vesicles reduced T‐cell proliferation and IFN‐gamma secretion (Kim et al. 2019). Blocking or genetically removing PD‐L1 from these vesicles restored T‐cell activity, supporting PD‐L1‐dependent immune suppression (Kim et al. 2019). In mouse models, administration of PD‐L1‐rich vesicles enhanced tumor growth, whereas PD‐L1 removal abrogated this effect (Kim et al. 2019).

Recent research extends this concept beyond static PD‐L1 cargo detection to include mechanisms underlying T‐cell dysfunction and immune checkpoint trafficking. Tumor extracellular vesicle‐derived PD‐L1 has been shown to promote T‐cell senescence through lipid‐metabolism reprogramming. Inhibition of extracellular vesicle synthesis or downstream lipid‐metabolic pathways reduces EV‐mediated T‐cell dysfunction in experimental models (Ma, Liu, et al. 2025). Additionally, Munc13‐4 has been identified as a regulator of PD‐L1 sorting and secretion via exosomes in breast tumor models. Deletion of Munc13‐4 reduces exosomal PD‐L1 secretion, enhances T‐cell‐mediated antitumor immunity, suppresses tumor growth, and improves the efficacy of immune checkpoint inhibitors (Liu, Liu, et al. 2025). Collectively, these studies suggest that targeting EV‐associated immune checkpoint trafficking is a promising approach, although current evidence remains preliminary and does not yet establish a confirmed clinical strategy.

This concept has been extended to epigenetic regulation of EV‐associated immune checkpoints. In gastric cancer, LSD1 was reported to increase PD‐L1 enrichment in exosome‐associated compartments without comparably increasing membrane‐bound PD‐L1 (Shen et al. 2022). Vesicles from LSD1‐expressing gastric cancer cells inhibited CD8+ T‐cell proliferation and function, whereas LSD1 knockout reduced vesicle‐associated PD‐L1 and restored anti‐tumor T‐cell responses in vitro and in vivo, with impaired tumor growth in immunocompetent but not T‐cell‐deficient mouse models (Shen et al. 2022). LSD1 expression also correlated negatively with CD8 expression and positively with PD‐L1 levels in patient‐derived gastric cancer tissues (Shen et al. 2022). These data support a model in which LSD1 promotes immune escape through EV‐associated PD‐L1, while leaving open the possibility that additional LSD1‐dependent mechanisms also contribute.

The regulation of EV‐associated checkpoint cargo is also influenced by tumor signaling state. In NSCLC models, PKCα was reported to regulate PD‐L1 expression and secretion in small EVs via p53‐dependent STAT1 signaling (Zhang, Liao, et al. 2025). A recent head and neck squamous cell carcinoma study further linked PD‐L1‐positive tumor‐derived EVs to CD8+ T‐cell terminal exhaustion and anti‐PD‐1 resistance, with EV‐associated PD‐L1 promoting BATF‐associated terminal‐exhaustion programs in CD8+ T cells (Fang et al. 2026). Together, these observations suggest that EV‐associated checkpoint ligands are not only passive biomarkers of tumor immune state but may also directly contribute to immune dysfunction and therapy resistance in selected models.

Inflammatory signaling can further intensify the immunosuppressive phenotype of tumor‐derived EVs. TLR4 activation in tumor cells has been reported to alter the composition and functional output of released EVs, increasing their capacity to suppress immune surveillance mechanisms (Domenis et al. 2019). Accordingly, TLR4‐driven inflammatory signaling represents a context‐dependent amplifier of tumor‐promoting EV communication (Domenis et al. 2019).

**FIGURE 5:**
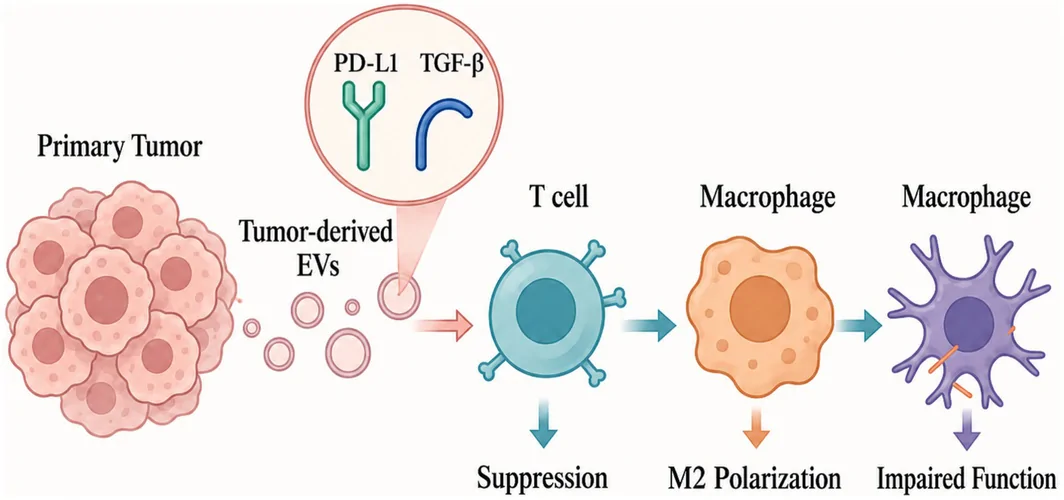
Tumor‐derived EVs contribute to immune evasion by carrying immunosuppressive cargo and reprogramming immune cells. Primary tumor cells release extracellular vesicles (EVs) that carry immunomodulatory molecules, such as PD‐L1 and TGF‐beta, when these molecules are present in the EV cargo. Tumor‐derived EVs suppress antitumor T‐cell activity and induce macrophage polarization toward an M2‐like, tumor‐supportive phenotype. This reprogramming of macrophages impairs antitumor function and facilitates the development of an immunosuppressive tumor microenvironment. Collectively, these EV‐mediated mechanisms diminish immune surveillance and promote tumor progression. This figure presents representative model‐based mechanisms and does not suggest a uniform immunosuppressive program for all tumor EVs.

Figure 5 summarizes representative immunosuppressive mechanisms mediated by tumor‐derived EVs, including delivery of PD‐L1 or TGF‐beta‐associated signals, modulation of T‐cell activity, and macrophage polarization toward tumor‐supportive phenotypes.

Macrophage reprogramming provides another example of EV‐mediated immune modulation. Exosome‐enriched preparations from metastatic osteosarcoma sublines (K7M3 and DLM8) reprogrammed alveolar macrophages toward an M2‐like phenotype, with increased IL‐10, TGF‐beta2, and CCL22 mRNA, reduced phagocytic capacity, and impaired tumoricidal function. Vesicles from non‐metastatic osteosarcoma cells lacked these effects, indicating that metastasis‐associated differences in vesicle activity exist. The same study implicated TGFB2 secretion in this immunosuppressive phenotype, as pharmacological inhibition of TGFB2 reversed the macrophage changes (Wolf‐Dennen et al. 2020).

More recent studies further connect EV‐associated RNA cargo with macrophage checkpoint regulation. Breast cancer‐derived small EVs enriched in miR‐106b‐5p and miR‐18a‐5p were reported to increase PD‐L1 expression in tumor‐associated macrophages through PTEN/AKT and PIAS3/STAT3 signaling, thereby linking EV‐miRNA transfer to macrophage polarization and immune evasion (Xu et al. 2023). In colorectal cancer, exosomal miR‐372‐5p was reported to promote immune escape via a PTEN/AKT/NF‐κB/PD‐L1 pathway that involves both tumor cells and macrophages, with downstream effects on CD8+ T‐cell activity (Wu, Xiao, et al. 2024). These examples reinforce the concept that EV‐mediated immunosuppression is not limited to direct checkpoint‐ligand transfer but can also occur through EV‐associated RNAs that reprogram checkpoint expression and immune‐cell phenotype.

Tumor‐derived EVs may also carry other immunosuppressive cargos, including TGF‐beta, IL‐10, prostaglandin E2, and immunoregulatory microRNAs, but the contribution of each cargo varies by tumor type and experimental system. Whiteside summarized multiple mechanisms by which tumor‐derived exosomes or EV‐enriched preparations can inhibit antitumor immunity, including promotion of suppressive myeloid phenotypes, regulatory T‐cell expansion, reduced cytotoxic T‐cell or natural killer cell activity, and impaired dendritic cell antigen presentation (Whiteside 2016). These conclusions support a broad immunosuppressive role for tumor‐derived EVs, but the underlying mechanisms remain dependent on the specific donor cell, recipient immune cell, cargo, and assay context (Whiteside 2016).

Overall, the strongest conclusion supported by the current evidence is that tumor‐derived EVs can modulate immune escape by transferring immune checkpoint ligands, regulatory RNAs, and soluble factor signals to T cells, macrophages, myeloid cells, and other immune compartments (Whiteside 2016; Yin et al. 2025). The most recent literature shifts the therapeutic interpretation from generic EV blockade toward mechanism‐specific strategies, such as disrupting PD‐L1 sorting or secretion, inhibiting EV‐induced metabolic or exhaustion programs in T cells, or targeting EV‐miRNA‐driven macrophage checkpoint regulation (Liu, Liu, et al. 2025; Ma, Liu, et al. 2025; Wu, Xiao, et al. 2024; Xu et al. 2023). However, these strategies remain largely preclinical and require tumor‐type‐specific validation, standardized EV characterization, functional add‐back or rescue controls, and careful assessment of off‐target effects before clinical translation (Welsh et al. 2024).

### 3.3 Stromal Reprogramming and Angiogenesis

The tumor microenvironment (TME) comprises cancer cells, fibroblasts, endothelial cells, pericytes, immune cells, and extracellular matrix components that support tumor growth, invasion, angiogenesis, and metastatic progression (Bremnes et al. 2011). Tumor‐derived EVs contribute to this stromal reprogramming, and CM‐based experiments have been particularly useful for testing direct effects of tumor‐cell‐derived vesicles on defined recipient populations. Cancer‐associated fibroblasts (CAFs) are pro‐tumorigenic stromal cells that secrete extracellular matrix components, growth factors, cytokines, and proteases, thereby facilitating tumor invasion, angiogenesis, and remodeling of the TME (Bremnes et al. 2011; Fiaschi et al. 2013). The interpretation of CM‐based effects requires consideration of the EV‐enrichment workflow, EV‐dose metric, recipient‐cell assay, and possible soluble or particulate co‐isolates, given that these variables can influence whether a stromal or vascular phenotype is attributable to EV‐associated cargo or to the broader CM milieu (Shekari et al. 2023; Welsh et al. 2024).

A well‐supported example of EV‐mediated stromal reprogramming is the conversion of fibroblasts or mesenchymal stromal cells into CAF‐like or myofibroblast‐like populations after exposure to tumor‐derived EVs. In breast cancer, tumor cell‐derived EVs reprogrammed adipose tissue‐derived mesenchymal stem cells toward a myofibroblast‐like phenotype associated with fibrosis, vascular remodeling, and tumor‐supportive stromal activity (Cho et al. 2012). Bladder cancer‐derived EVs induced fibroblast‐to‐myofibroblast transition through cargos including TGF‐beta and fibronectin, thereby enhancing invasion‐supportive stromal behavior (Ringuette Goulet et al. 2018). In head and neck cancer, EV‐associated maspin released by irradiated tumor cells was implicated in modulating the stromal response, including the suppression of fibroblast chemotaxis (Dean et al. 2016).

Additional mechanistic studies demonstrate that specific EV cargos can induce fibroblast activation, depending on the tumor context. In hepatocellular carcinoma, EV‐associated miR‐1247‐3p derived from highly metastatic cells activates fibroblasts by targeting B4GALT3 and engaging beta1‐integrin‐NF‐kappaB signaling, which promotes a pro‐metastatic stromal phenotype (Fang et al. 2018). Together, these findings support the concept that tumor‐derived EVs can remodel stromal cell behavior through defined protein and RNA cargo, although the relevant recipient cell type, cargo, and functional outcome vary by tumor context.

Data from colorectal cancer extend this fibroblast‐activation axis. SW480‐derived exosomes were reported to activate stromal fibroblasts, increase CAF‐associated markers and cytokines, and promote VEGF‐A‐related stromal output partly through miR‐224‐mediated regulation of PHLPP1/2 and Akt signaling (Khaloozadeh et al. 2024). This aspect supports a cargo‐linked model of EV‐driven stromal reprogramming, although the conclusion remains tied to the specific CRC cell model, EV preparation dose, and fibroblast assay conditions used in that study (Khaloozadeh et al. 2024; Welsh et al. 2024).

Recent bidirectional models also show that EV‐activated stromal cells can feed back on cancer‐cell fitness. In lung cancer models, tumor‐derived EV‐activated CAFs promoted metastatic competence by transferring mitochondrial DNA to tumor cells, restoring mitochondrial function and increasing resistance to oxidative stress (Zhou, Qu, et al. 2024). This finding broadens the interpretive framework from one‐way tumor‐to‐stroma signaling to reciprocal tumor‐stroma metabolic support, while still requiring model‐specific interpretation (Welsh et al. 2024; Zhou, Qu, et al. 2024).

A hypoxic tumor state can further reshape EV‐mediated stromal responses. In colorectal cancer, hypoxia‐induced tumor exosomes have been shown to activate normal tissue‐associated fibroblasts toward a cancer‐associated fibroblast (CAF) phenotype more effectively than normoxic exosomes. HIF1A‐AS2 present in the hypoxic exosomal cargo is associated with miR‐33 sequestration, activation of HIF‐1alpha/Notch1/ERK signaling pathways, and increased expression of VEGF, MMP‐7, and MMP‐9. Xenograft studies demonstrate that these exosomes promote CAF infiltration, angiogenesis, extracellular matrix reorganization, and tumor growth (Han et al. 2026). These findings illustrate how culture oxygen tension can influence extracellular vesicle (EV)‐mediated stromal and vascular programming; however, current evidence is limited to colorectal cancer models and requires validation in additional systems (Han et al. 2026; Shekari et al. 2023).

Apart from fibroblasts and mesenchymal stromal cells, tumor‐derived EVs can also modulate melanocyte plasticity. Melanoma cell‐derived EVs were reported to drive phenotypic switching in primary melanocytes via paracrine and autocrine mechanisms, including MAPK activation and regulation of EMT‐related microRNAs such as let‐7i, miR‐191, and let‐7a (Xiao et al. 2016). Transcriptomic analyses further showed that melanocytes exposed to melanoma‐derived EVs displayed enrichment of inflammatory and leukocyte‐chemotaxis pathways, whereas lung cancer‐derived EVs induced a distinct transcriptional response enriched for DNA replication and mitotic programs (Xiao, Li, et al. 2020).

**FIGURE 6:**
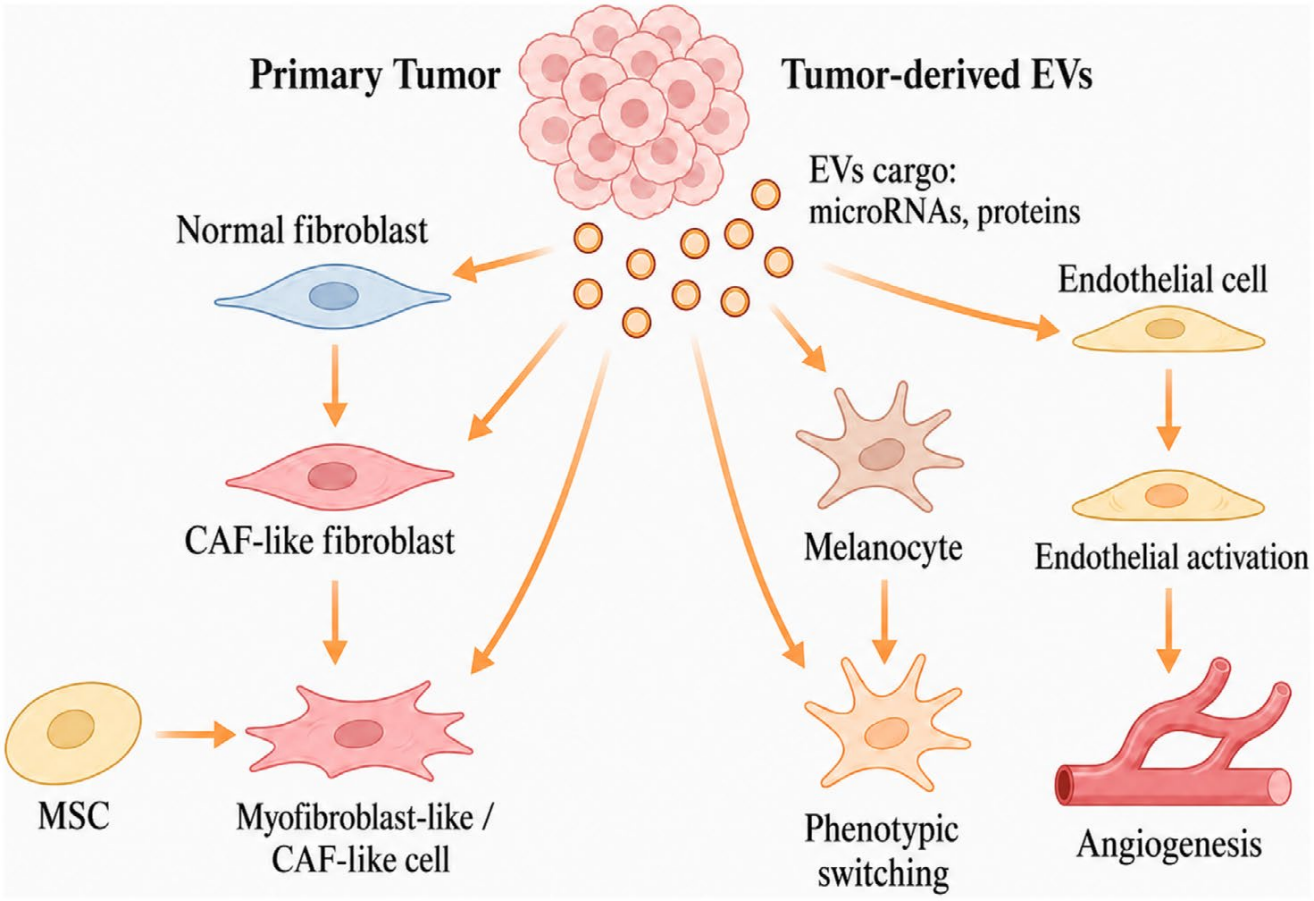
Tumor‐derived EVs promote stromal reprogramming and angiogenic remodeling within the tumor microenvironment. Primary tumor cells release EVs containing regulatory cargo, such as microRNAs and proteins, which modulate various non‐malignant recipient cell populations. These EVs facilitate the transition of normal fibroblasts to cancer‐associated fibroblast (CAF)‐like phenotypes and reprogram mesenchymal stromal/stem cells (MSCs) into myofibroblast‐like or CAF‐like cells, supporting stromal remodeling and tumor‐promoting microenvironmental alterations. Additionally, tumor‐derived EVs induce phenotypic changes in melanocytes and activate endothelial or vascular‐associated pathways, contributing to angiogenesis in specific models. Collectively, these EV‐mediated processes reshape the stromal landscape to favor tumor progression. This figure synthesizes model‐based evidence and does not imply that all tumor‐derived EV preparations elicit identical stromal or angiogenic responses.

Overall, these observations suggest that tumor‐derived EVs can reprogram neighboring non‐malignant cells in a tumor‐type‐ and recipient‐cell‐dependent manner, contributing to stromal remodeling, angiogenesis, and the establishment of a permissive tumor microenvironment. Figure 6 illustrates how tumor‐derived EVs can modulate fibroblasts, mesenchymal stromal cells, melanocytes, endothelial cells, and other recipient populations, thereby promoting remodeling of the tumor microenvironment and cancer progression.

These observations do not support the conclusion that EV effects are consistent across all cancer types. Rather, the findings indicate that the response to tumor‐derived EVs depends on both the donor tumor type and the recipient cell context. For instance, melanoma‐derived EVs modulate inflammatory and leukocyte chemotaxis pathways in melanocytes, while lung cancer‐derived EVs predominantly enhance DNA replication and mitotic pathways (Xiao, Li, et al. 2020). This distinction is important when applying CM‐based findings to the diverse in vivo tumor microenvironment. For instance, endothelial dysfunction and vascular inflammation can promote cancer progression and metastasis by dysregulating adhesion, permeability, and inflammatory signaling linked to NF‐kappaB or STAT3, creating a non‐EV vascular background against which EV effects must be interpreted (Malhab et al. 2021).

Tumor‐derived EVs also influence endothelial cell function, but their cargo and cellular targets differ by tumor type. In glioma, hypoxia‐induced EV‐associated miR‐106a‐5p was not linked to endothelial angiogenesis but primarily to temozolomide resistance through PTEN inhibition and PI3K/Akt activation (Wu et al. 2022). In epithelial ovarian cancer, EV‐associated miR‐940 promoted M2 macrophage polarization and tumor growth‐related phenotypes (Chen et al. 2017). A more direct endothelial example is provided by hypoxic leukemia cell‐derived exosomes enriched in miR‐210, which enhanced endothelial tube formation under hypoxic stress (Tadokoro et al. 2013).

EV‐associated vascular remodeling is closely connected to metastatic progression. As noted above, melanoma‐derived exosome‐enriched preparations induced vascular leakiness at pre‐metastatic sites, thereby priming the vasculature for tumor‐cell extravasation (Peinado et al. 2012). Studies targeting EV‐release regulators, including Rab27A‐centered pathways, further support the concept that vesicle secretion can influence angiogenesis and tumor growth, although these interventions may also affect non‐EV cellular processes and therefore require cautious interpretation (Sun et al. 2018).

Consistent with this vascular‐remodeling framework, melanoma‐derived EVs have been linked to pro‐inflammatory signaling in metastatic microenvironments and to cytokine or chemokine changes that can favor vascular permeability (Gener Lahav et al. 2019). More broadly, tumor EVs have been associated with VEGF‐related and inflammatory mediators involved in neovascularization, but these effects are tumor‐ and context‐dependent (Wang et al. 2016; Xue et al. 2017).

EV‐associated miRNAs can also potentiate angiogenesis and vascular remodeling. CRC‐derived exosomal miR‐25‐3p suppressed tight‐junction proteins, including ZO‐1, occludin, and claudin‐5, and upregulated VEGFR2 in endothelial cells by targeting KLF2 and KLF4, thereby enhancing vascular permeability and neovascularization. Systemic administration of CRC‐derived exosomal miR‐25‐3p increased vascular leakiness and metastatic colonization of liver and lung tissues in mice (Zeng et al. 2018). Elevated circulating exosomal miR‐25‐3p in metastatic CRC patients supports its potential as a biomarker, but its clinical utility remains to be validated in larger, independent cohorts.

Recent endothelial‐focused studies extend this miRNA axis. In colorectal cancer, exosomal miR‐320d enhanced endothelial migration and angiogenic capacity by downregulating GNAI1 and activating JAK2/STAT3‐VEGFA signaling, with in vivo evidence of increased tumor growth, vascularization, and liver metastasis (Wu, Zhang, et al. 2024). Another colorectal cancer study reported that cancer‐secreted exosomal miR‐1825 was transferred to vascular endothelial cells, suppressed ING1, activated TGF‐beta/Smad2/Smad3 signaling, and promoted angiogenesis and liver metastasis in experimental models (Sun et al. 2025). Conversely, in bladder cancer, the SLC7A7‐miR‐152‐3p‐FGFR3 study illustrates that EV‐associated miRNAs may also restrain angiogenic signaling depending on cargo direction and donor‐cell state; reduced transfer of anti‐angiogenic miR‐152‐3p relieved inhibition of FGFR3‐dependent endothelial responses (Cao et al. 2025). Together, these examples support endothelial reprogramming as a recurrent EV‐mediated process, but not a single conserved pathway across tumor types (Cao et al. 2025; Sun et al. 2025; Wu, Zhang, et al. 2024).

In summary, controlled CM‐based systems have shown that tumor‐derived EVs can remodel stromal and vascular compartments by activating fibroblasts, modulating melanocytes or mesenchymal stromal cells, increasing endothelial permeability, and promoting angiogenesis in selected models. These effects highlight the role of EV‐mediated tumor‐stroma crosstalk, although the magnitude and direction of the response depend on tumor type, culture conditions, EV‐isolation strategy, recipient‐cell identity, and the presence of soluble co‐isolates. The most informative recent studies strengthen this view by combining EV transfer with cargo perturbation, recipient‐cell pathway analysis, and in vivo validation, but causal attribution still requires dose normalization and controls for soluble co‐isolates, especially when CM‐derived EV‐enriched preparations are used (Shekari et al. 2023; Welsh et al. 2024).

### 3.4 Therapeutic Resistance and Survival Advantages

Therapeutic resistance provides another context in which CM‐based transfer experiments have been informative. EV‐enriched preparations from drug‐resistant cancer cells can transfer resistance‐associated cargo to drug‐sensitive recipient cells, although the strength of causal inference depends on EV‐depletion, add‐back, cargo‐perturbation, and dose‐normalization controls. Mechanistically, EVs may contribute to resistance through drug‐efflux proteins, anti‐apoptotic proteins, and regulatory RNAs, including microRNAs that target tumor‐suppressor or apoptosis‐related pathways (Li et al. 2021; Ren et al. 2024).

These mechanisms represent context‐specific resistance routes instead of a single shared EV program across cancers, because EV cargo, donor‐cell state, recipient‐cell phenotype, drug class, isolation workflow, and co‐isolated soluble material can all influence the observed resistance phenotype (Ren et al. 2024; Welsh et al. 2024).

P‐glycoprotein (MDR1/P‐gp) transfer illustrates the need for precise source attribution. A recent chemotherapy‐exposure model in breast cancer implicated serum‐derived EVs in the acquisition of multidrug resistance by transferring glycosylated, functionally active P‐gp (Zhou, Hong, et al. 2025). In that study, EV‐associated glycosylated P‐gp escaped proteasomal degradation and retained drug‐pumping capacity, while chemotherapy‐related vascular effects facilitated EV access to the tumor microenvironment (Zhou, Hong, et al. 2025). Because this evidence derives from serum‐derived EVs, it supports the broader concept of EV‐associated P‐gp transfer but does not provide direct evidence for CM‐derived tumor EV‐mediated multidrug resistance.

EV‐associated microRNAs provide additional resistance mechanisms. Reviews and primary studies have linked miR‐155, miR‐21, and other EV‐associated microRNAs to altered apoptosis, survival signaling, drug uptake, or drug‐response pathways in recipient cancer cells (Li et al. 2021; Zhao et al. 2024). In gastric cancer, paclitaxel‐resistant MGC‐803R cells released exosome‐enriched preparations carrying miR‐155‐5p, which induced EMT and chemoresistance in sensitive MGC‐803S cells through suppression of GATA3 and TP53INP1 via direct 3′ UTR targeting (Wang et al. 2019).

**FIGURE 7:**
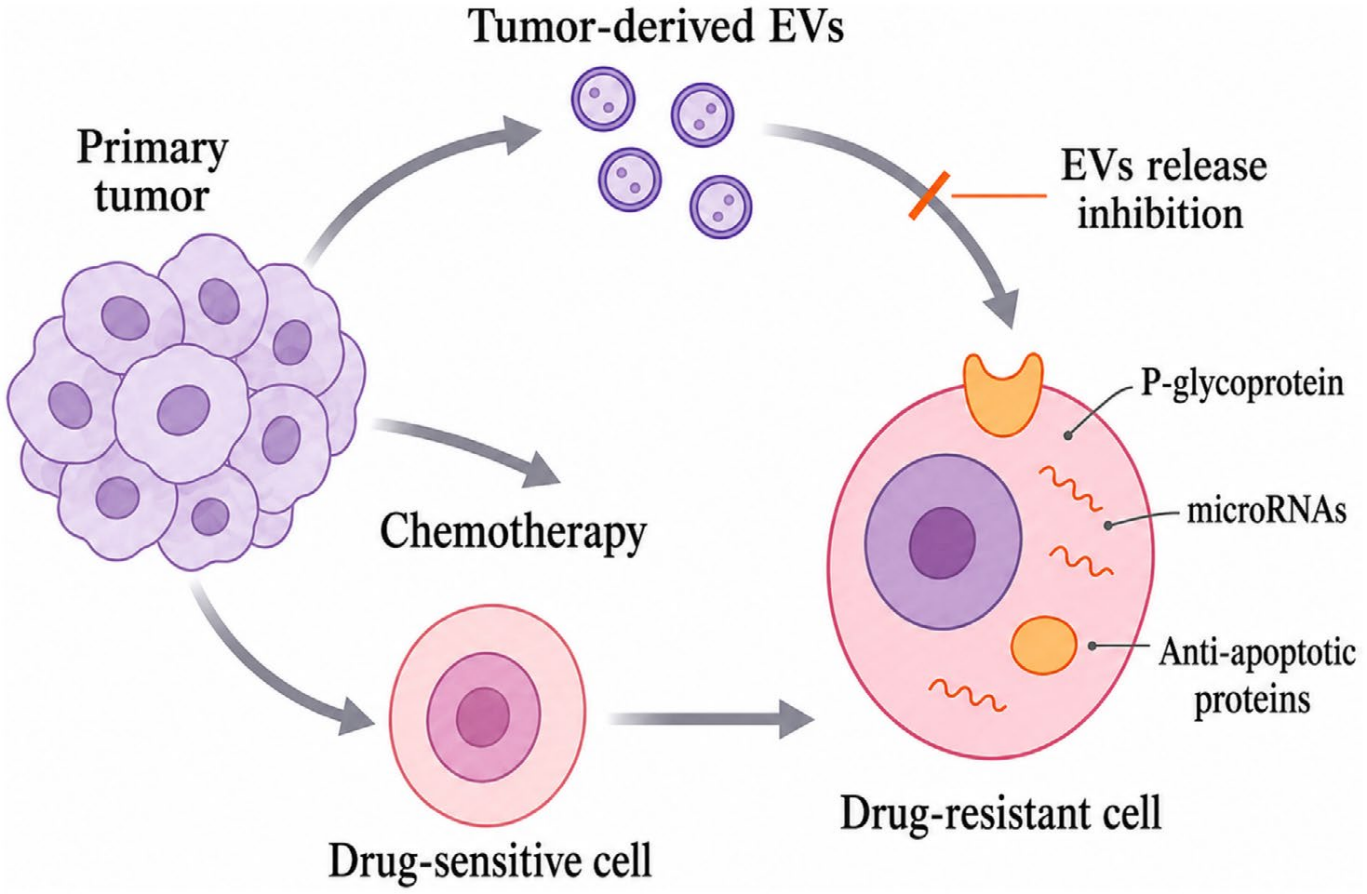
Tumor‐derived EVs contribute to chemoresistance by transferring resistance‐associated cargo between cells. Primary tumor cells secrete EVs that facilitate the transfer of resistance‐promoting molecules to recipient cancer cells. Under chemotherapeutic pressure, drug‐sensitive cells can develop a drug‐resistant phenotype through EV‐mediated delivery of cargo such as P‐glycoprotein (P‐gp), regulatory microRNAs, and anti‐apoptotic proteins. These EV‐associated components enhance cell survival and diminish responsiveness to chemotherapy. The figure further illustrates EV release inhibition as a potential strategy to disrupt resistance‐promoting intercellular communication and enhance therapeutic efficacy; however, such interventions require rigorous evaluation of specificity and potential off‐target effects. The figure presents selected resistance mechanisms identified in defined experimental systems and does not suggest that all tumor‐derived EVs confer resistance or that EV‐release inhibition has been clinically validated.

Similarly, exosome‐enriched preparations from cisplatin‐resistant oral squamous cell carcinoma (OSCC) cell lines transferred resistance traits to parental cells, reducing DNA‐damage responses and increasing survival after cisplatin exposure. The functional effect was traced to EV‐associated miR‐21, which downregulated PTEN and PDCD4, and in vivo xenograft experiments supported the role of resistant cell‐derived vesicles in modulating drug response (Liu et al. 2017). In gastric cancer, miR‐21 was also upregulated in cisplatin‐resistant SGC7901/DDP cells, where miR‐21 overexpression reduced apoptosis and enhanced survival, whereas miR‐21 silencing restored cisplatin cytotoxicity through PTEN‐Akt signaling (Yang et al. 2013). These findings support a recurrent, but context‐dependent, role for miR‐21 in chemoresistance. Figure 7 illustrates how tumor‐derived EVs may spread resistance‐associated cargo such as P‐glycoprotein, regulatory microRNAs, and anti‐apoptotic signals between cancer cells.

Recent work extends EV‐mediated resistance beyond microRNAs to include long non‐coding RNAs as cargo. In bladder cancer, exosomes from cancer stem‐like or gemcitabine‐resistant cell populations transferred LUCAT1 to recipient cells, enhanced stemness and gemcitabine chemoresistance, and mechanistically linked LUCAT1 to HMGA1 mRNA stabilization through IGF2BP2‐dependent regulation (Zhan et al. 2025). This example connects EV cargo transfer, cancer stemness, and drug resistance, but it should still be interpreted as tumor‐ and model‐specific evidence and cannot be extrapolated to a generalized lncRNA‐resistance pathway (Welsh et al. 2024; Zhan et al. 2025).

Targeted‐therapy resistance provides a complementary example. In EGFR‐mutant lung adenocarcinoma, hypoxia‐induced exosomal LUCAT1 was reported to promote osimertinib resistance by stabilizing c‐MET and activating AKT/mTOR and ERK signaling; c‐MET inhibition partly restored drug sensitivity in experimental models (Du et al. 2025). This finding supports EV‐associated cargo as a potential source of actionable recipient‐cell vulnerabilities, although clinical utility requires independent validation in patient cohorts and treatment contexts (Du et al. 2025).

In general, EVs can contribute to chemoresistance by mediating the intercellular transfer of resistance‐associated cargo, drug sequestration or extrusion, and the activation of survival pathways in stressed tumor cells. However, these mechanisms do not constitute a single universal pathway, because the dominant EV‐dependent resistance mechanism varies by tumor type, drug, EV source, and assay context (Ren et al. 2024; Wu et al. 2022).

In addition to traditional chemotherapy resistance, tumor‐derived EVs have been linked to resistance to targeted therapies, antibody treatments, and immunotherapies. Current evidence suggests that EVs primarily sequester or transfer immunomodulatory and resistance‐related molecules, rather than providing a universal resistance mechanism across tumor types. For example, EV‐associated PD‐L1 can inhibit T‐cell activity, potentially reducing the effectiveness of PD‐1/PD‐L1 inhibitors (Kim et al. 2019), while CD20‐positive EVs released by B‐cell lymphomas can bind to anti‐CD20 antibodies, decreasing their availability (Aung et al. 2011). This antibody sequestration enables lymphoma cells to evade antibody‐mediated destruction, ultimately compromising therapeutic efficacy (Aung et al. 2011).

Recent evidence in immune‐oncology further refines the PD‐L1 concept. Tumor extracellular vesicle‐derived PD‐L1 has been reported to promote T‐cell senescence and suppression through lipid‐metabolism reprogramming, supporting a mechanistic link between EV‐associated immune checkpoints and impaired antitumor immunity (Ma, Liu, et al. 2025). This finding strengthens the rationale for considering EV‐PD‐L1 in the context of immunotherapy resistance, but by itself, it does not establish EV‐PD‐L1 blockade as a clinically validated therapeutic strategy (Ma, Liu, et al. 2025; Welsh et al. 2024).

Alongside antibody sequestration, lymphoma‐derived EVs may also reflect and modulate disease‐relevant molecular and immune programs. In DLBCL, EVs carried molecular information related to aberrant transcriptional programming and genomic alterations, supporting their broader relevance in lymphoma biology (Rutherford et al. 2018). Ling et al. reported that DLBCL‐derived exosome‐enriched preparations promoted macrophage polarization toward a protumor M2‐like phenotype and altered cytokine release, supporting a role for lymphoma EVs in immune remodeling (Ling et al. 2020). Shao et al. further showed that DLBCL‐derived EV‐associated ENO2 modulated tumor‐associated macrophage polarization through metabolic reprogramming, thereby promoting DLBCL progression (Shao et al. 2024). These findings indicate that lymphoma‐derived EVs may contribute to therapy resistance not only by intercepting antibody‐based therapies but also by reinforcing an immunosuppressive microenvironment that supports tumor persistence during treatment.

More broadly, tumor‐derived EVs can help create a protective niche for cancer cells under therapeutic pressure by promoting survival signaling, reducing apoptosis, altering DNA‐damage responses, or carrying decoy targets that sequester therapeutic agents. Whiteside highlighted the role of tumor‐derived exosomes in immune suppression and drug resistance, supporting their relevance to cancer therapy (Whiteside 2016). Nevertheless, causal attribution requires controlled transfer experiments, EV‐depleted and add‐back conditions, and cargo‐specific perturbation, because CM from resistant cells also contains soluble mediators and other co‐isolates that may contribute to the observed phenotype. Dose normalization and controls for soluble or particulate co‐isolates are also needed when resistance phenotypes are attributed to EV‐enriched fractions and not to whole CM or mixed extracellular material (Welsh et al. 2024).

These mechanistic observations motivate strategies aimed at disrupting EV biogenesis, release, or cargo transfer, but such approaches remain largely preclinical and require careful specificity assessment. Neutral sphingomyelinase‐dependent ceramide generation has been implicated in exosome formation (Datta et al. 2017; Kong et al. 2015). GW4869 has been reported to suppress exosome release and dampen inflammatory responses in experimental models (Essandoh et al. 2015). The same compound has also been used to reduce exosome production in non‐cancer inflammatory settings, highlighting its role as an experimental tool rather than a clinically approved anticancer treatment (Essandoh et al. 2015). In castration‐resistant prostate cancer cells, inhibition of Ras/Raf/ERK signaling by manumycin A reduced exosome biogenesis and secretion (Datta et al. 2017).

Several studies further suggest that aggressive cancer phenotypes, including melanoma and breast cancer models, can display increased EV or exosome release associated with invasive behavior (Hoshino et al. 2013; Singh et al. 2014). However, increased vesicle output represents a context‐dependent feature of aggressive tumor biology, not proof that EV release alone determines metastatic potential. Associations between high vesicle output, disease progression, and therapeutic targeting of EV secretion have been reported (Ohshima et al. 2014; Zhao et al. 2022). For example, suppression of RAB27A‐related extracellular vesicle (EV) transport pathways has been associated with decreased vesicle secretion and reduced metastatic behavior in cancer models (Wang et al. 2024b). In colorectal cancer, Apatinib has been evaluated for its anti‐tumor properties and its ability to reduce tumor‐derived exosome secretion, indicating that inhibition of EV release may enhance anticancer therapy in specific contexts (Zhao et al. 2022).

Inhibiting extracellular vesicle (EV) uptake represents a second conceptually promising strategy; however, this area is less developed and more context‐dependent. Scavenger receptor‐related pathways have been implicated in EV uptake and systemic EV trafficking (Singh et al. 2014; Verweij et al. 2019). For example, SR‐B1 targeting has been reported to inhibit cellular exosome uptake in model systems (Plebanek et al. 2015). However, the available evidence does not establish Stabilin‐2 neutralization as a validated anticancer EV‐uptake blockade strategy; rather, it supports treating receptor‐mediated uptake as a mechanistic lead requiring tumor‐specific validation. Literature on liver sinusoidal endothelial scavenger functions and Stabilin‐2/HARE biology provides biological plausibility for receptor‐mediated EV handling, but not direct proof of therapeutic blockade of tumor‐derived EV signaling (Bhandari et al. 2021; Harris and Baker 2020). In multiple myeloma models, inhibition of tumor EV uptake by bone marrow stromal cells suppressed stromal‐cell responses (Zheng et al. 2019), whereas engineered EV approaches have also been explored to redirect pathological inflammatory or immune pathways (Wang et al. 2023).

Overall, EV‐targeting strategies have the potential to disrupt tumor‐promoting communication, immune suppression, and resistance transfer; however, current evidence is predominantly preclinical and mechanistically diverse. Therefore, EV release, cargo transfer, and uptake‐blockade studies should be viewed as mechanistic and translational hypotheses derived from CM and in vivo models, not as established clinical interventions. In this context, CM studies are essential because they enable a precise definition of donor cells, experimental perturbations, EV‐enriched fractions, EV‐depleted controls, and add‐back experiments, which are often not feasible in patient biofluids.

## 4 EV‐Associated Biomarkers in Cancer Diagnosis

Early and accurate cancer diagnosis is critical for improving patient outcomes; however, conventional diagnostic methods are often limited by suboptimal sensitivity, specificity, invasiveness, infrequent sampling, and tumor heterogeneity. As a result, EV‐associated biomarkers have emerged as promising candidates for minimally invasive cancer detection and monitoring (Kumar 2025). EVs can protect molecular cargo within a lipid bilayer and can be recovered from accessible biofluids, such as blood, urine, and saliva; therefore, they represent a biologically informative platform for liquid biopsy research (Kumar 2025). Tumor‐derived EVs may contain cargo that reflects the molecular phenotype of the parental tumor cells, including proteins and microRNAs with diagnostic, prognostic, or treatment‐monitoring value (Kumar 2025).

Recent cancer‐focused EV reviews support this translational interest, while emphasizing that EV‐associated biomarkers function as complementary liquid‐biopsy analytes, not as automatic replacements for established imaging, tissue biopsy, circulating tumor DNA, circulating tumor cells, or conventional protein‐marker workflows (Greening et al. 2025; Shan et al. 2025). At the same time, EV‐based diagnostics remain technically demanding. Bulk EV profiling can obscure vesicle heterogeneity, whereas single‐EV and onco‐EV detection technologies may improve analytical resolution by identifying rare tumor‐associated subpopulations. However, tumor‐derived EVs may represent only a small fraction of total circulating EVs in biofluids, requiring high‐throughput measurements, targeted enrichment, and strict control of false‐positive and false‐negative signals (Imanbekova et al. 2025; Liu, Chen, and Yu 2025).

Clinical translation also requires analytical standardization. Pre‐analytical variables, EV separation strategy, purity, co‐isolate burden, marker selection, normalization, and reporting practices can influence the apparent performance of biomarkers. Candidate EV biomarkers must meet the same evidentiary standards as other liquid‐biopsy tools, including reproducible analytical performance, independent validation, clinically meaningful thresholds, and demonstrated added value over existing diagnostic or monitoring approaches (Shan et al. 2025; Welsh et al. 2024).

**FIGURE 8:**
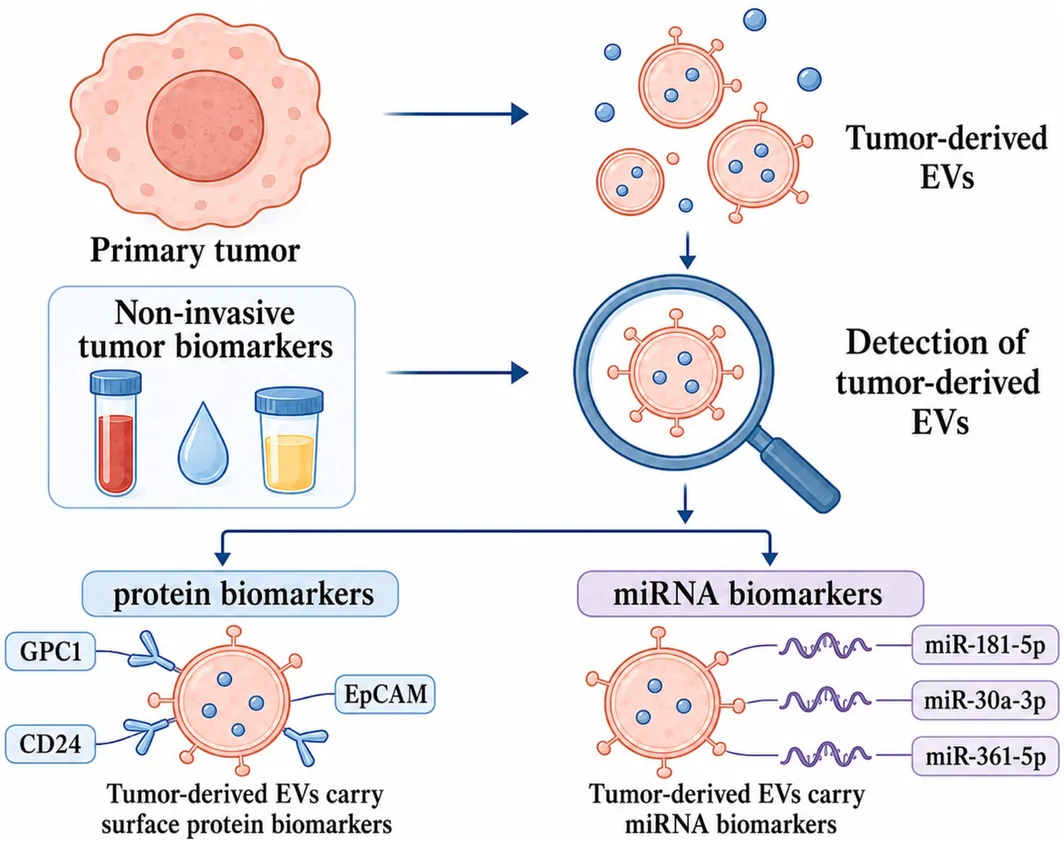
Tumor‐derived EVs as minimally invasive sources of biomarkers for cancer diagnosis. Primary tumor cells secrete extracellular vesicles (EVs) into accessible biofluids, enabling detection and profiling of EV‐associated cargo through liquid biopsy. Tumor‐derived EVs contain both surface and intraluminal candidate biomarkers that partially represent the molecular phenotype of the originating tumor cells. These biomarkers include proteins such as GPC1, EpCAM, and CD24, as well as EV‐associated microRNAs (miRNAs) including miR‐181‐5p, miR‐30a‐3p, and miR‐361‐5p. Profiling these EV‐associated proteins and miRNAs facilitates minimally invasive cancer detection, tumor characterization, disease monitoring, and prognostic assessment. The clinical utility of these biomarkers, however, relies on standardized EV isolation and characterization protocols, effective control of co‐isolated non‐vesicular material, analytically validated assays, and prospective validation in clinically relevant patient cohorts.

Accordingly, Figure 8 presents an overview of the profiling of tumor‐derived EVs as sources of candidate protein and microRNA biomarkers for cancer detection, tumor characterization, disease monitoring, and prognostic assessment. Although EV‐associated biomarkers are biologically informative and technically promising, their clinical utility depends on the development of harmonized workflows and prospective validation in clinically relevant cohorts (Greening et al. 2025; Shan et al. 2025; Welsh et al. 2024).

### 4.1 EV‐Associated miRNAs as Candidate Diagnostic Biomarkers

EV‐associated miRNAs are widely investigated as minimally invasive candidate biomarkers for early cancer detection, disease monitoring, and prediction of treatment response. Their relative stability in circulation and detectability in accessible body fluids support their diagnostic appeal, while reported cancer‐associated miRNA signatures suggest possible associations with tumor type, stage, and biological behavior (Behulová et al. 2023). In parallel, EV‐associated miRNAs can influence treatment‐relevant cellular processes, including proliferation, apoptosis, and drug resistance, although mechanistic effects must be distinguished from their use as circulating biomarkers (Guo, Wang, et al. 2020). Because EV‐miRNA signatures are highly sensitive to biofluid source, EV‐enrichment strategy, RNA extraction method, normalization approach, and statistical model, biomarker interpretation remains assay‐ and cohort‐specific unless independently validated under harmonized pre‐analytical and analytical conditions (Welsh et al. 2024).

In addition to their role in static diagnosis, EV‐associated miRNAs are under investigation as indicators of disease progression and therapeutic response. Alterations in EV‐associated miRNA profiles have been correlated with disease progression, metastasis, and treatment response, indicating their potential for longitudinal monitoring. However, clinical implementation will require prospective validation and standardized analytical workflows (Kumar 2025). From a biological perspective, cancer‐cell‐derived EVs may facilitate communication within the tumor microenvironment by transferring oncogenic or tumor‐suppressive miRNAs to recipient cells. These mechanistic functions are distinct from the use of EV‐associated miRNAs as biomarkers in patient biofluids (Kumar 2025).

Recent disease‐specific studies illustrate this transition from candidate discovery toward risk‐stratification panels. In neuroblastoma, plasma small‐EV miRNA sequencing followed by validation identified dysregulated miRNAs, including miR‐150‐5p, miR‐142‐5p, miR‐30b‐5p, miR‐320a‐3p, miR‐30b, and miR‐342‐3p, with diagnostic potential and partial discrimination of MYCN‐amplified disease; however, the study is not a broadly generalizable EV‐miRNA test but remains a tumor‐specific validation example (Zhou, Wang, et al. 2025).

EV‐associated miRNAs may also help distinguish cancer subtypes. In a next‐generation sequencing study of non‐small cell lung cancer (NSCLC), tumor‐derived exosomal miRNAs were profiled to discriminate adenocarcinoma from squamous cell carcinoma. The investigators identified adenocarcinoma‐associated miR‐181‐5p, miR‐30a‐3p, miR‐30e‐3p, and miR‐361‐5p, together with squamous cell carcinoma‐associated miR‐10b‐5p, miR‐15b‐5p, and miR‐320b. In that cohort, the miRNA panel demonstrated promising discriminative performance, supporting its candidacy for NSCLC subtype stratification and early detection, although routine clinical implementation remains to be established (Jin et al. 2017).

A separate investigation assessed the diagnostic utility of serum exosomal miRNAs in early‐stage gastric cancer. The study identified miR‐92b‐3p, miR‐146b‐5p, miR‐9‐5p, and let‐7g‐5p as candidate non‐invasive biomarkers for gastric cancer detection. These results indicate that circulating extracellular vesicle (EV)‐associated miRNA panels may complement conventional diagnostic methods. However, larger and independent validation studies are necessary prior to routine clinical implementation (Tang et al. 2020).

Subsequent research on gastric cancer has advanced this approach by developing a diagnostic model that uses five miRNA pairs derived from glycosylated EVs. The independent validation performance reported in this study highlights the potential of EV‐miRNA panels. Nevertheless, the findings are currently platform‐specific and require further external validation and comparison with established diagnostic pathways (Wang et al. 2025).

A meta‐analysis conducted by Wong and Chen assessed the diagnostic and prognostic potential of exosomal biomarkers across various cancer types. The study demonstrated favorable pooled diagnostic performance across diverse malignancies. For prognostic evaluation, the authors examined 50 biomarkers in 4797 patients from 42 studies, identifying significant associations with overall survival, disease‐free survival, and recurrence‐free survival. These results indicate that extracellular vesicle (EV)‐associated biomarkers are promising candidates; however, interpretation should consider assay heterogeneity, variability in study design, and the necessity for external validation (Wong and Chen 2019).

Pancreatic cancer further illustrates the balance between innovation and methodological restraint. A multiethnic radiogenomics study integrated radiologic features with plasma EV‐derived miRNA profiling for early diagnosis and molecular subtyping, whereas a 2025 systematic review of EV‐derived miRNAs in pancreatic ductal adenocarcinoma concluded that clinical translation remains limited by inconsistent isolation methods, incomplete EV validation, small cohorts, and limited external validation (Patel et al. 2025; Xu, Shi, et al. 2025).

Overall, EV‐associated miRNAs are best presented as candidate biomarkers or components of multiparametric panels, and should not yet be framed as stand‐alone clinical tests. Their translation will require standardized EV enrichment and characterization, transparent reporting of co‐isolates, pre‐specified cut‐offs, independent prospective validation, and demonstration of added clinical value over existing diagnostic or monitoring strategies (Patel et al. 2025; Welsh et al. 2024).

### 4.2 Protein Biomarkers and Cancer Type‐Specific EV Signatures

In addition to microRNAs, EV‐associated proteins are being investigated as diagnostic and prognostic biomarkers across several cancer types (De Giorgis et al. 2024). EVs can display surface and intraluminal proteins related to the cell of origin, tumor phenotype, adhesion state, immune checkpoint signaling, and pathway activation (Pham et al. 2025). When reproducibly detected in patient biofluids, these proteins may support tumor classification, disease monitoring, or risk stratification; however, clinical utility depends on standardized EV enrichment, multi‐method characterization, analytical validation, and independent clinical validation (De Giorgis et al. 2024; Pham et al. 2025). Surface‐protein signals may reflect the captured extracellular vesicle (EV) subset, the biofluid background, and the analytical platform. Consequently, interpretation of protein biomarkers remains assay‐ and cohort‐specific unless EV specificity, cell‐of‐origin attribution, and independent validation are established (Welsh et al. 2024).

For breast cancer, Risha et al. identified glucose transporter 1 (GLUT‐1), glypican‐1 (GPC‐1), and ADAM10 among exosomal membrane proteins with potential diagnostic relevance; these proteins were elevated in EV preparations from breast cancer samples, supporting their candidacy without establishing definitive diagnostic performance (Risha et al. 2020). More recently, plasma‐derived EV proteomics in triple‐negative breast cancer (TNBC) revealed disease‐associated protein profiles and identified Histone H2A as a candidate TNBC‐associated marker present before surgery; this finding expands the protein‐biomarker discussion but still requires larger external validation before clinical implementation (Tamarindo et al. 2025).

In urinary cancers, Wang et al. reported increased expression of several proteins in bladder cancer cell‐derived EVs, including MUC1, integrins beta1 and alpha6, CD36, CD44, CD10, 5T4, basigin, and CD73. The same study described distinctive protein signatures, including TM256, in urinary EVs from prostate cancer patients, suggesting the value of EV protein profiling in genitourinary malignancies while leaving prospective clinical validation as a necessary next step (Wang et al. 2020).

In lung cancer, pleural effusion vesicle proteomics in NSCLC identified 912 proteins, many of which differed from those in non‐malignant conditions and included EGFR pathway‐associated proteins, supporting the relevance of EV proteomics for tumor biology and biomarker discovery (Park et al. 2013). A recent plasma EV proteomic study in lung squamous cell carcinoma (LUSC) used nanomaterial‐based EV enrichment, mass spectrometry, and machine‐learning models to identify EV‐associated protein signatures capable of distinguishing LUSC from benign lung disease and supporting prognostic assessment; importantly, the authors also noted demographic imbalance, possible non‐EV co‐isolates, and the need for EV‐specific quantification of candidate proteins (Ma, Zhao, et al. 2025).

In colorectal cancer, several candidate EV‐associated biomarkers, including carcinoembryonic antigen (CEA), EGFR, MAPK4, proliferating cell nuclear antigen (PCNA), and keratin 18, have been identified and may facilitate tumor characterization and biomarker discovery (Xiao, Zhong, et al. 2020). Proteins associated with metastatic phenotypes, such as MET, S100A8, S100A9, and tenascin C, have also been detected in colorectal cancer‐derived vesicle preparations, thereby linking EV‐associated cargo to invasion and pre‐metastatic niche biology (Suwakulsiri et al. 2019). In 2024, an EV‐based plasma proteomics workflow that integrated rapid EV capture, SP3 preparation, data‐independent acquisition mass spectrometry (DIA‐MS), and machine learning quantified over 800 EV‐associated proteins. This approach utilized a 10‐protein panel to classify adenomatous polyps and early‐stage colorectal cancer in an independent single‐blind validation cohort, achieving an accuracy of 89.3%. These data reinforce the translational potential of EV protein panels and underscore the necessity for standardized workflows prior to routine screening implementation (Zhang, Gao, et al. 2024).

In ovarian cancer, vesicle preparations from malignant ascites were reported to contain CD24 and epithelial cell adhesion molecule (EpCAM) (Runz et al. 2007), whereas claudin‐4‐containing exosomes were detected in plasma from ovarian cancer patients in a pilot study, supporting their potential use as circulating candidate biomarkers while requiring broader validation (Li et al. 2009). Targeted proteomics of plasma EVs identified MUC1 as part of early high‐grade serous carcinoma (HGSC) biomarker combinations with ROC‐AUC values greater than 0.90 in discovery/validation analyses (Cooper et al. 2024). A subsequent circulating sEV proteomic study identified fallopian tube‐lineage and STIC‐associated candidate biomarkers, including MYL6, GSTP1, TTYH3, PRDX6, MUC1, MYH14, and PTGS1, and reported that a four‐protein panel (MUC1, MYL6, TTYH3, and GSTP1) distinguished early‐stage HGSOC from healthy controls with high reported performance in the study cohort (Rayamajhi et al. 2026). These results are promising, although they remain candidate‐panel evidence pending multicenter validation and harmonized pre‐analytical workflows.

Thyroid cancer‐derived EV preparations have also been associated with epithelial‐to‐mesenchymal transition (EMT)‐related biology. EV‐associated proteins such as SRC, talin 1 (TLN1), integrin beta‐2 (ITGB2), and calpain small subunit 1 (CAPNS1) have been linked to EMT‐associated features and enhanced cellular motility (Luo et al. 2018).

Pancreatic cancer‐derived EVs have been investigated for enrichment in glypican‐1 (GPC1), a surface proteoglycan proposed as a candidate marker for pancreatic cancer. Earlier studies detected GPC1‐positive EVs in patient blood and explored their use for early diagnosis and disease monitoring (Buscail et al. 2019; Melo et al. 2015). A more recent study combined analyses of extracellular vesicular GPC1 mRNA and protein for pancreatic cancer diagnosis and prognosis, supporting the value of paired nucleic acid/protein EV readouts while reinforcing that GPC1 remains a candidate biomarker requiring context‐specific validation rather than a universally definitive pancreatic cancer test (Li, Chiang, et al. 2024). In addition, EV‐associated integrin patterns have been linked to organ‐specific metastatic behavior, with alpha6beta4 and alpha6beta1 associated with lung tropism and alphavbeta5 associated with liver tropism in experimental models (Hoshino et al. 2015).

Recent single‐vesicle methodologies demonstrate that interpreting protein biomarkers should not depend exclusively on bulk extracellular vesicle (EV) lysates. Techniques such as single‐EV imaging and high‐plex surface profiling enable the identification of EV subpopulations characterized by distinct combinations of surface proteins and intraluminal cargo, including EpCAM‐positive and PD‐L1‐positive EV subsets. These advances enhance biological specificity and treatment‐monitoring capabilities, although they also introduce greater assay complexity (Wu et al. 2025).

In summary, EV‐associated protein biomarkers represent candidate signatures and components of multiparametric panels, not uniformly established clinical tests. Representative examples of cancer‐associated EV biomarkers and their reported diagnostic or prognostic relevance are summarized in Table 2. Their translation into routine oncology diagnostics will require standardized isolation and characterization, EV‐specific quantification instead of measurement of total soluble protein, harmonized proteomic or immunoassay platforms, clinically meaningful thresholds, prospective multicenter validation, and transparent reporting of co‐isolates and pre‐analytical variables (De Giorgis et al. 2024; Pham et al. 2025). These requirements are consistent with current EV reporting guidance and are particularly important when bulk EV‐protein measurements are used to infer tumor‐specific signals (Welsh et al. 2024).

**TABLE 2:** Selected cancer‐associated EV biomarkers and their reported diagnostic or prognostic relevance across tumor types.

Study references	Cancer type	EV‐associated biomarkers/platform	Reported relevance
Risha et al. (2020)	Breast cancer	GLUT‐1, GPC‐1, ADAM10	Candidate breast cancer EV protein markers
Tamarindo et al. (2025)	Triple‐negative breast cancer	Plasma EV proteomic profiles; Histone H2A	Candidate TNBC‐associated plasma EV protein marker; requires external validation.
Wang et al. (2020)	Bladder and prostate cancer	MUC1, integrins beta1/alpha6, CD36, CD44, CD10, 5T4, basigin, CD73, TM256	Urinary EV protein profiling in genitourinary malignancies
Ma, Zhao, et al. (2025)	Lung squamous cell carcinoma	Plasma EV protein signatures; TUBB3, RPS7, RPLP1 among diagnostic candidates	LUSC versus benign lung disease discrimination and prognostic modeling; limitations include possible co‐isolates
J. Zhang, Gao, et al. (2024)	Colorectal cancer	10‐protein EV panel from rapid‐capture/DIA‐MS workflow	Polyp and early‐stage CRC classification in an independent single‐blind validation cohort
Cooper et al. (2024); Rayamajhi et al. (2026)	High‐grade serous ovarian cancer	MUC1; MYL6, GSTP1, TTYH3, PRDX6, MUC1, MYH14, PTGS1; four‐protein panel MUC1/MYL6/TTYH3/GSTP1	Early HGSC candidate panels linked to fallopian tube‐lineage and STIC‐associated EV proteins
Li, Chiang, et al. (2024)	Pancreatic cancer	EV‐associated GPC1 mRNA and GPC1 protein	Paired EV nucleic‐acid/protein analysis for PDAC diagnosis/prognosis; candidate marker requiring contextual validation
Wu et al. (2025)	Pan‐cancer/immunotherapy monitoring platform	Single‐EV profiling of EpCAM+ and PD‐L1+ EV subsets with intraluminal cargo readout	Illustrates the need for single‐EV resolution to address biomarker heterogeneity

*Note:* This table summarizes selected studies reporting protein‐based and paired protein/nucleic acid extracellular vesicle (EV)‐associated candidate biomarkers identified across various human cancers. Each entry specifies the tumor type, representative EV‐associated components, and the reported diagnostic or prognostic relevance, including early detection, subtype differentiation, disease monitoring, or metastasis‐related profiling. As the listed biomarkers were identified using diverse sample sources, isolation workflows, analytical platforms, and validation designs, they should be considered candidate biomarkers rather than uniformly established clinical tests. These findings underscore the potential of EV‐associated proteins as liquid biopsy analytes for tumor profiling and emphasize the need for analytical standardization, reproducibility, and independent clinical validation.

## 5 Therapeutic Perspectives Arising From EV Biology in Oncology

Therapeutic applications of EVs, including engineered exosome‐enriched preparations, reflect a broader translational perspective grounded in EV biology rather than solely in studies of CM‐derived tumor EVs. EVs are under investigation both as intrinsic biological products and as delivery systems for small‐molecule drugs, proteins, mRNAs, microRNAs, siRNAs, and other therapeutic cargos (Cheng and Kalluri 2023). However, the purported advantages of EVs over synthetic nanocarriers, such as lipid nanoparticles, depend on the source, cargo, administration route, dose, disease context, and comparator; therefore, these benefits must be demonstrated experimentally and should not be presumed to be inherent to the class (Cheng and Kalluri 2023). This approach aligns with quality and safety recommendations for EV therapeutic products, which emphasize source control, product characterization, potency, and safety evaluation prior to clinical translation (Takakura et al. 2024).

Accordingly, two related yet evidentially distinct domains are delineated. Studies of CM‐derived tumor EVs are instrumental in identifying actionable vesicle‐associated programs, cargo‐transfer mechanisms, and recipient‐cell pathways. In contrast, the development of engineered EV medicines requires independent product definition, manufacturing controls, pharmacology, toxicology, and regulatory validation before substantiating therapeutic activity, safety, or platform advantage (Cheng and Kalluri 2023). Within this product‐development framework, EV‐drug evaluations must specify source material, identity and purity criteria, impurity and co‐isolate profiling, dose metrics, potency assays, biodistribution, and nonclinical safety in a product‐specific manner (Xu, Jin, et al. 2025).

Recent literature on EV‐drug development reinforces this cautious perspective by shifting focus from general EV therapeutic potential to product‐specific development requirements. For clinical translation, EV‐based products must have a clearly defined mechanism of action, controlled starting material and cell source, identity and purity specifications, impurity and co‐isolate profiling, potency assays, dose metrics, biodistribution data, scalable and GMP‐compatible production processes, storage stability, release criteria, and an indication‐appropriate nonclinical safety package (Takakura et al. 2024; Xu, Jin, et al. 2025).

Regulatory and clinical guidance publications indicate that EV therapies constitute an evolving product class, not a fully established clinical platform. A 2025 clinical guidance document reported that pharmaceutical products with EVs as the primary active ingredient have not yet received regulatory approval, and emphasized risk profiling, manufacturing process quality, EV verification, and effectiveness assessment as essential requirements for clinical application (Terai et al. 2025). Regulatory reviews also highlight fragmented jurisdictional pathways and ongoing challenges in quality control, safety evaluation, efficacy demonstration, product heterogeneity, and oversight of EV or secretome preparations (Verma and Arora 2025).

In oncology, this distinction is particularly significant, as tumor‐derived EVs may be viewed either as biological mediators that reveal targetable cancer communication pathways or as conceptual templates for engineered therapeutic platforms. Recent cancer‐focused reviews support the therapeutic relevance of EVs as drug‐delivery, vaccine, and immunotherapy platforms, but also underscore that clinical implementation is constrained by technical, analytical, and translational barriers (Greening et al. 2025). Consequently, therapeutic interpretation in oncology needs explicit linkage to the EV source, engineering strategy, cargo, target indication, and comparator platform and cannot be generalized to all EVs as a class (Greening et al. 2025; Xu, Jin, et al. 2025).

For these reasons, the following subsections present EV therapeutics as a promising yet technically demanding area of translational research. Selected EV platforms may achieve therapeutic utility only when their source, cargo loading, targeting, potency, biodistribution, scalability, reproducibility, storage, and regulatory quality attributes are defined with the same rigor as those of other advanced biologic products.

### 5.1 Engineered EVs as Drug and Nucleic‐Acid Delivery Platforms

Engineered EVs, including platforms described as exosomes or exosome‐enriched preparations, have been developed to deliver small‐molecule drugs, proteins, and nucleic‐acid cargos, including siRNAs, shRNAs, mRNAs, and other therapeutic RNAs (Liang, Duan, et al. 2021). This delivery concept is attractive because EV membranes can protect labile cargo and mediate cell entry; nevertheless, therapeutic performance depends on EV source, loading method, route of administration, payload chemistry, and disease context, and cannot be inferred from EV identity alone (Takakura et al. 2024; Xu, Jin, et al. 2025).

Preclinical drug‐loading studies demonstrate both the potential and the limitations of this platform. In multidrug‐resistant cell models, paclitaxel‐loaded exosome preparations enhance cytotoxicity compared with free paclitaxel, indicating partial circumvention of P‐glycoprotein‐mediated efflux under defined experimental conditions (Luan et al. 2017). Exosome‐delivery studies and reviews focused on brain cancer have also examined paclitaxel‐loaded or chemotherapeutic extracellular vesicle (EV) platforms in glioblastoma‐relevant contexts. Nevertheless, these findings remain primarily preclinical and necessitate direct comparison with established nanocarriers, dose‐matched controls, and comprehensive safety assessments before translational superiority can be established (Basyoni et al. 2025).

In addition to passive cargo loading, EVs can be engineered to enhance tumor targeting or tissue specificity. Donor cells may be genetically modified to express targeting ligands on EV surfaces, while isolated vesicles can be chemically functionalized after purification with targeting moieties or other surface modifications (Luan et al. 2017). Although these strategies may improve tumor‐cell recognition, they can also affect EV composition, biodistribution, immunogenicity, clearance, and manufacturing reproducibility. Consequently, robust evidence for targeting efficacy requires the inclusion of uptake, biodistribution, potency, dose–response, and safety data (Takakura et al. 2024; Xu, Jin, et al. 2025).

Recent receptor‐defined engineering studies illustrate the transition from passive vesicle loading to active tumor targeting. In a 2025 Nature Communications study, a CD63‐fused anti‐HLA‐G nanobody was used to generate HLA‐G‐targeted EVs that preferentially entered HLA‐G‐positive tumor cells and delivered doxorubicin, temozolomide, or carboplatin in triple‐negative breast cancer, glioblastoma, and ovarian cancer models, including patient‐derived primary tumor cells (Shie et al. 2025). This example supports the feasibility of antigen‐guided EV delivery; however, it should be interpreted as preclinical evidence that requires validation of antigen heterogeneity, liver and spleen accumulation, off‐target uptake, safety, scalable production, and batch reproducibility (Shie et al. 2025; Xu, Jin, et al. 2025).

Overall, engineered EVs should be seen as flexible biological delivery tools rather than naturally superior nanocarriers. In oncology, the most substantiated conclusions are case‐specific and comparator‐aware. The selection of EV source, cargo‐loading method, surface‐engineering strategy, and administration route must be tailored to the intended tumor target and supported by cross‐validated characterization, release criteria, payload quantification, functional potency assays, biodistribution studies, and nonclinical safety evaluations (Takakura et al. 2024; Xu, Jin, et al. 2025). Product performance requires evaluation against relevant non‐EV or synthetic nanocarrier comparators using matched payloads, administration routes, doses, and efficacy or safety endpoints and should not be inferred solely from EV engineering (Takakura et al. 2024; Xu, Jin, et al. 2025).

### 5.2 Functional Cargo and Oncogenic Pathway Targeting

A key proof‐of‐concept for oncogene‐directed EV therapy comes from engineered exosomes derived from fibroblast‐like mesenchymal cells, loaded with siRNA or shRNA targeting KRAS ^G12D^, and tested in pancreatic cancer models. These engineered vesicles, termed iExosomes, expressed CD47, showed prolonged circulation, and suppressed tumor growth in preclinical pancreatic cancer models (Kamerkar et al. 2017). This evidence supports the feasibility of EV‐mediated oncogene silencing in a defined preclinical context; however, it should not be generalized as proof that all EV platforms are intrinsically superior to lipid nanoparticles or other RNA‐delivery systems. This comparator‐aware interpretation is consistent with recent analyses comparing EVs and lipid nanoparticles for nucleic‐acid delivery, which emphasize formulation, biodistribution, transfection performance, clinical maturity, and manufacturing constraints, without supporting class‐wide superiority (Bader et al. 2024).

The iEXPLORE Phase I study constitutes a significant recent clinical update on engineered exosomes carrying KRAS^G12D^‐specific siRNA in advanced metastatic pancreatic ductal adenocarcinoma. This first‐in‐human, open‐label, single‐center, non‐randomized dose‐escalation trial enrolled patients with metastatic PDAC harboring KRAS^G12D^ mutations following failure of multiple prior therapies. The primary outcomes assessed safety, tolerability, and target engagement, while definitive efficacy was not within the scope of this early‐phase investigation (Kalluri et al. 2025). In this context, iExoKrasG12D achieved early safety and tolerability endpoints and demonstrated pharmacodynamic signals, including KRASG12D DNA reduction, phospho‐ERK suppression, and increased intratumoral CD8+ T cells in selected analyses (Kalluri et al. 2025). These results should be regarded as preliminary evidence of feasibility, safety, and target engagement for engineered extracellular vesicle (EV)‐mediated RNA delivery, rather than as definitive proof of clinical efficacy or superiority over other nucleic acid delivery systems. Given the early‐phase, non‐randomized design, these pharmacodynamic and immunological findings should be considered hypothesis‐generating and specific to the product studied.

Recent preclinical studies indicate a transition in extracellular vesicle (EV) engineering from passive cargo loading to active surface targeting. For example, a 2025 Nature Communications study engineered HEK293T cells to produce EVs displaying an anti‐HLA‐G nanobody‐chimeric CD63 fusion protein, thereby enhancing targeting of HLA‐G‐positive tumor cells. After loading with doxorubicin, temozolomide, or carboplatin, these alpha‐HLA‐G EVs were evaluated in triple‐negative breast cancer, glioblastoma, and ovarian cancer models, including in vitro and in vivo systems and patient‐derived primary tumor‐cell models (Shie et al. 2025). This study demonstrates the feasibility of surface‐engineered EVs that integrate tumor‐recognition modules with chemotherapeutic agents; however, these findings remain preclinical and should be interpreted as evidence of delivery‐platform feasibility rather than established clinical benefit.

EV‐mediated drug delivery has also been investigated using tumor‐cell‐derived vesicle preparations. In prostate cancer models, paclitaxel‐loaded EVs showed increased in vitro cytotoxicity compared with the free drug, with delivery primarily via endocytic uptake. However, interpretation differed across EV subpopulations, and these results provide preclinical evidence of delivery feasibility rather than definitive proof of clinical efficacy (Saari et al. 2015).

Collectively, the evidence discussed above supports a graded evidentiary framework: preclinical proof‐of‐concept for functional cargo delivery, early clinical feasibility for KRAS^G12D^‐directed iExosomes, and emerging preclinical evidence for surface‐targeted chemotherapeutic EV platforms. In parallel, EV‐based drug development requires product‐specific evaluation of the source cell, engineering strategy, cargo‐loading method, identity, purity, potency, dose metric, biodistribution, release criteria, stability, and nonclinical safety. A 2025 overview of EV drug development highlighted the ongoing technical heterogeneity of EV therapeutics, underscoring the need for robust quality control and nonclinical evaluation frameworks to enable successful clinical translation (Xu, Jin, et al. 2025). Functional cargo delivery and oncogenic pathway targeting are promising translational strategies. However, these approaches remain product‐specific and require further development into uniformly validated therapeutic classes. In the case of nucleic‐acid delivery, comparator‐aware evaluation should address payload encapsulation and release, target engagement, extrahepatic biodistribution, immunogenicity, repeat‐dosing feasibility, and manufacturability (Bader et al. 2024; Xu, Jin, et al. 2025).

### 5.3 Targeting Tumor‐Promoting EV Programs and Hypoxia‐Driven Communication

Hypoxia is a major feature of many solid tumors and can reshape EV release, cargo composition, and biological output (Niazi and Ghafouri‐Fard 2025). Within hypoxic tumor microenvironments, EV‐mediated communication can involve cancer, endothelial, stromal, and immune compartments, contributing, in a context‐dependent manner, to angiogenesis, vascular permeability, metastatic niche formation, macrophage polarization, and therapy resistance (Niazi and Ghafouri‐Fard 2025). These EV‐mediated effects intersect with the broader therapeutic challenge of tumor vascular remodeling, in which abnormal angiogenic signaling, fragile and hyperpermeable vessels, inefficient perfusion, adaptive resistance to anti‐angiogenic therapy, and delivery‐platform limitations shape the response to vascular‐targeted interventions (Giordo et al. 2022). Hypoxia‐driven EV communication should therefore be considered a therapeutically relevant, but context‐dependent, process rather than a uniform therapeutic target. A recent systematic review focused on hypoxia‐induced EV‐associated non‐coding RNAs in preclinical animal cancer models similarly supports links with angiogenesis, metastatic behavior, and therapy response, while reinforcing that therapeutic interpretation remains model‐dependent (Afonso et al. 2025).

In pancreatic cancer models, hypoxia‐induced exosome‐enriched preparations from PANC‐1 and BxPC‐3 cells were reported to promote vasculogenic mimicry by activating the HIF‐1alpha/LARS1/mTOR axis, whereas Rg3 inhibited this process in vitro and in vivo (Zhao et al. 2025). This example supports the concept that tumor‐promoting EV programs can be pharmacologically modulated without providing evidence that EV‐directed therapies are already clinically established in this setting.

Recent studies have expanded this concept to include vascular remodeling and the formation of pre‐metastatic niches. In esophageal squamous cell carcinoma, hypoxia‐induced exosomal circ‐ZNF609 has been shown to promote angiogenesis and disrupt the vascular barrier through mechanisms involving the miR‐150‐5p/VEGFA and HuR/ZO‐1 axes. This finding links hypoxic extracellular vesicle (EV) cargo to increased vascular permeability and metastatic niche formation (Mao et al. 2024). In lung adenocarcinoma, hypoxia‐induced exosomal miR‐671‐3p derived from H1975 cells has been found to promote angiogenesis by regulating the KLF2‐VEGFR2 axis. These results suggest that hypoxic EV‐associated microRNAs may serve as potential targets for anti‐angiogenic or anti‐metastatic therapies (Liu, Wang, et al. 2025). These findings reinforce the idea that hypoxia‐induced EV cargo can functionally reprogram endothelial responses, although therapeutic translation will require validation across models, tumor types, and clinically relevant EV‐isolation workflows. These vascular examples identify candidate recipient‐cell pathways without yet establishing them as validated therapeutic targets because the evidence remains restricted to specific tumor models and EV cargo contexts (Afonso et al. 2025).

Hypoxia‐driven EV communication may also play a role in treatment resistance. In EGFR‐mutant lung adenocarcinoma, hypoxia‐induced exosomal LUCAT1 was shown to disseminate osimertinib resistance by stabilizing c‐MET and activating downstream AKT/mTOR and ERK signaling (Du et al. 2025). In the same study, inhibition of c‐MET signaling partly restored drug sensitivity in experimental models, supporting a therapeutically actionable link between hypoxia‐induced EV cargo and targeted‐therapy resistance (Du et al. 2025). This evidence extends the therapeutic rationale beyond direct EV inhibition to include combined strategies targeting EV‐induced recipient‐cell pathways, including VEGF/VEGFR2, mTOR, c‐MET, and other context‐specific signaling nodes. Therefore, translational prioritization requires EV‐specific transfer, cargo perturbation, dose‐normalized functional assays, and comparison with non‐EV explanations before a pathway is advanced as an EV‐targeted intervention (Welsh et al. 2024; Xu, Jin, et al. 2025).

In summary, hypoxia‐driven EV programs should be considered as modifiable components of tumor–stroma communication rather than as a single therapeutic target. The most effective translational strategy is to avoid assuming broad clinical efficacy of EV blockade and instead prioritize identifying tumor‐specific EV cargoes and recipient‐cell pathways that may be inhibited, monitored, or integrated with existing anti‐angiogenic, anti‐metastatic, or targeted therapies. This measured approach is consistent with current EV reporting guidelines and recent analyses of EV‐drug development (Welsh et al. 2024; Xu, Jin, et al. 2025).

### 5.4 EV‐Based Vaccines, Immune Modulation, and Translational Constraints

Outside their established role in drug delivery, EVs have been explored as immunomodulatory platforms that can present tumor antigens, deliver immune‐activating cargo, or reshape tumor‐immune interactions (Greening et al. 2025). Early‐phase clinical studies evaluated dendritic cell‐derived exosomes loaded with tumor antigens as cancer vaccines in metastatic melanoma and reported antigen‐specific immune responses in some patients (Escudier et al. 2005). These studies established clinical feasibility, but their therapeutic activity was limited; contemporary DC‐EV vaccine strategies therefore focus on improving antigen selection and loading, incorporating immune‐stimulatory cargo or adjuvant immunotherapies, optimizing EV production and delivery protocols, and engineering EVs with tailored immunological properties (Shpigelman and Rao 2025). This updated vaccine‐oriented literature supports optimization strategies for antigen display, immune‐stimulatory cargo, dosing, and combination design, but it does not establish EV vaccines as clinically validated anticancer therapies.

Recent immune‐oncology EV programs have moved beyond antigen presentation toward local and cell‐directed immune pharmacology. Dendritic cell‐derived EVs engineered to display interleukin‐12 (IL‐12) and anti‐CTLA‐4 on their surface enhanced Th1 responses and reversed exhausted CD8+ T‐cell phenotypes in the tumor microenvironment in preclinical models (Chen et al. 2025). In a distinct lung‐cancer strategy, inhalable EVs loaded with IL‐12 mRNA delivered locally and promoted systemic antitumor immunity in preclinical models, addressing the challenge of cytokine exposure outside the tumor site (Liu et al. 2024). These examples support cytokine‐directed EV engineering as a preclinical immune‐pharmacology strategy, but not yet as an established clinical modality. Therefore, route‐specific delivery, local immune activation, and systemic cytokine exposure require joint assessment when cytokine‐loaded EVs are proposed for cancer immunotherapy.

A second major direction involves the delivery of innate immune agonists. ExoSTING, an engineered extracellular vesicle (EV) carrying cyclic‐dinucleotide STING agonists, demonstrated increased potency compared to free cyclic dinucleotides and promoted local Th1 responses, CD8+ T‐cell recruitment, and systemic antitumor immunity in preclinical models, while reducing systemic inflammatory cytokine exposure (Jang et al. 2021). More recently, bacterial EVs loaded with a STING agonist synergistically enhanced antitumor immunity and inhibited melanoma and colon tumor growth in preclinical models (Lotvall et al. 2025). Collectively, these studies indicate that EV formulation can modulate innate immune activation and local exposure. However, safety, biodistribution, dose window, and comparability with non‐EV formulations remain critical considerations for clinical translation. Furthermore, these innate‐immune EV systems require direct comparison with non‐EV STING agonist formulations under dose‐matched conditions before platform‐level advantages can be established.

**FIGURE 9:**
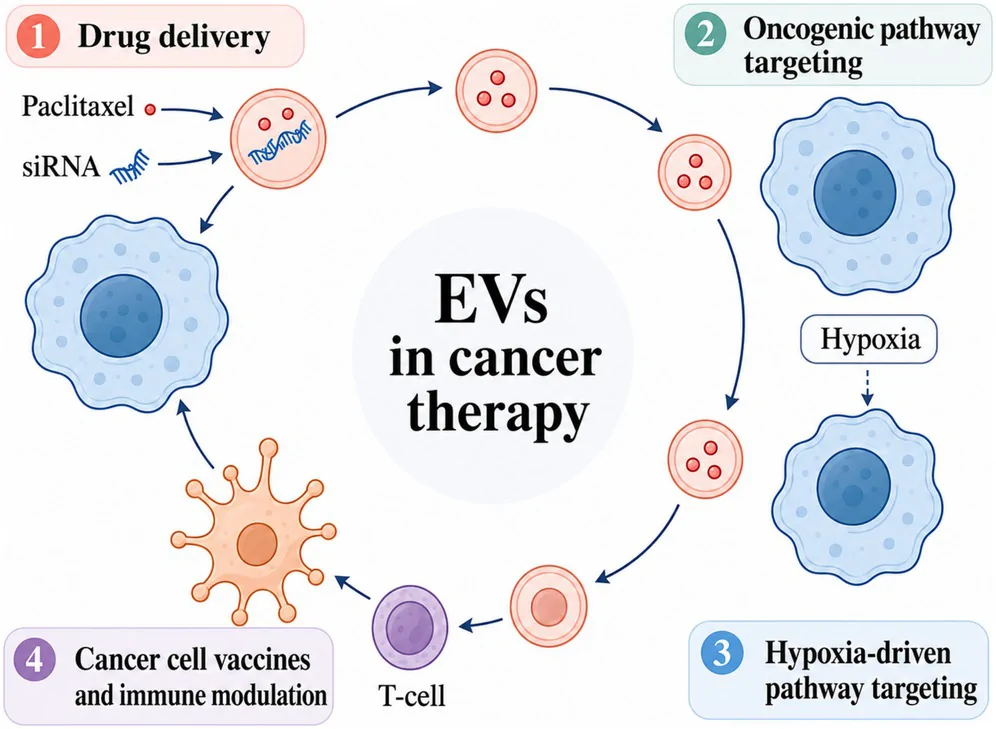
Therapeutic opportunities arising from EV biology and engineered EV platforms in cancer. EV‐based therapeutic strategies encompass engineered vesicle systems for drug and nucleic acid delivery, including loading with paclitaxel or siRNA, as well as interventions targeting oncogenic and hypoxia‐driven pathways. Additionally, EV platforms are being investigated for cancer vaccination and immune modulation, with a particular focus on enhancing antitumor T‐cell responses. These applications demonstrate the translational potential of EVs in oncology while underscoring that therapeutic development requires rigorous control over EV source, cargo loading, targeting specificity, potency, scalability, storage stability, batch consistency, and product reproducibility. The figure provides a translational framework and does not suggest that EV‐based immunotherapies are standardized or clinically established for all cancer indications.

In summary, EV‐based therapeutic strategies in oncology encompass engineered vesicle systems for the delivery of small molecules and nucleic acids, EV vaccines or immunomodulatory EVs, and platforms designed to modulate oncogenic, hypoxia‐driven, or innate immune pathways. The supporting evidence remains inconsistent across different indications and is primarily derived from preclinical models, with limited early clinical experience. As a result, EV therapeutics represent a promising yet technically challenging area for translation. Clinical progress depends on rigorous control of EV source, cargo loading, targeting specificity, potency, dose metric, biodistribution, safety, scalability, storage, batch consistency, product reproducibility, and regulatory quality attributes (Greening et al. 2025; Verma and Arora 2025; Xu, Jin, et al. 2025). Recent clinical guidance and regulatory analyses also emphasize the importance of risk profiling, manufacturing‐process quality, EV verification, effectiveness assessment, product‐specific quality attributes, and harmonized regulatory standards as prerequisites for therapeutic translation (Terai et al. 2025; Verma and Arora 2025; Xu, Jin, et al. 2025). Figure 9 provides an overview of these therapeutic opportunities and the principal translational constraints.

Taken together, recent literature shifts the therapeutic rationale from viewing EVs as intrinsically superior nanocarriers to considering them as engineered biologic products whose value must be demonstrated on a case‐by‐case basis. The strongest defensible conclusions are that engineered EVs can deliver drugs, nucleic acids, cytokine programs, or innate immune agonists in selected preclinical models; that KRAS^G12D^‐directed EV‐siRNA delivery has entered early clinical testing in pancreatic cancer; and that further translation will depend on defined and reproducible product attributes, mechanism‐linked potency assays, validated biodistribution, safety monitoring, and comparative performance against existing nanomedicine platforms (Greening et al. 2025; Kalluri et al. 2025; Verma and Arora 2025; Xu, Jin, et al. 2025).

## 6 Challenges and Limitations of CM‐Derived EV Research

Limitations inherent to CM‐derived tumor EV research impose significant constraints on data interpretation. These include issues related to analytical purity and co‐isolation, CM‐specific pre‐analytical variability, physiological incompleteness, functional causality, and translational readiness. Such constraints differentially impact mechanistic, biomarker, and therapeutic interpretations, necessitating careful consideration before extrapolating CM‐derived findings to patient biofluids, animal models, or EV‐based products (Shekari et al. 2023; Welsh et al. 2024; Xu, Jin, et al. 2025).

Despite substantial progress in extracellular vesicle (EV) biology and translational research, multiple challenges persist that hinder the interpretation and clinical implementation of EV‐based diagnostics and therapeutics. The primary limitation is methodological: CM‐derived preparations are typically enriched for EVs, but are not analytically pure, and may include soluble proteins, serum‐derived particles, protein aggregates, lipoprotein‐like particles, and other non‐vesicular co‐isolates. Ultracentrifugation remains a widely used method for enriching EVs from CM; however, it is time‐consuming, requires large sample volumes, and may compromise recovery or vesicle integrity due to high centrifugal forces (Doyle and Wang 2019; Zhang et al. 2020). Ultrafiltration and size‐exclusion chromatography (SEC) provide faster or gentler processing, but these methods also entail trade‐offs in recovery, purity, and the co‐isolation of size‐overlapping contaminants (Shu et al. 2021; Zhang et al. 2020). This methodological limitation imposes an important interpretive constraint: without multiparametric characterization and explicit assessment of co‐isolates, the observed biological effects cannot be confidently attributed to EVs, because they may also arise from mixed extracellular material (Welsh et al. 2024).

Immunoaffinity‐based methods can increase enrichment of selected EV subpopulations by targeting accessible surface antigens such as CD9, CD63, or CD81, but their performance depends on marker expression, epitope accessibility, antibody specificity, and recovery efficiency (Fernandes et al. 2025; Li et al. 2017; Welsh et al. 2024). Moreover, no single marker uniquely distinguishes endosome‐derived exosomes from other EV subtypes, complicating subtype assignment, purity assessment, and cross‐study comparison (Thery et al. 2018; Welsh et al. 2024). Accordingly, immunoaffinity capture represents marker‐defined enrichment or detection of selected EV subpopulations, not total EV recovery, analytical purity, or proof of exosomal origin (Fernandes et al. 2025; Welsh et al. 2024).

Current reporting standards have partially addressed these issues. MISEV2018 and MISEV2023 emphasize operational EV terminology, transparent reporting of pre‐analytical variables, complementary characterization, and cautious interpretation of EV subtype identity (Thery et al. 2018; Welsh et al. 2024). However, adoption remains uneven, and technical reproducibility remains a bottleneck for clinical translation. Earlier foundational exosome biology studies remain useful for background, but they are not adequate substitutes for current minimal reporting recommendations (Théry et al. 2002). The appropriate benchmark for current CM‐derived EV studies is therefore MISEV‐based operational terminology, transparent reporting of culture and separation variables, and complementary characterization (Thery et al. 2018; Welsh et al. 2024).

Scalability remains a significant challenge. Recent developments, such as three‐dimensional culture systems, tangential‐flow or nanosieve‐based workflows, and chromatography‐based purification strategies, aim to enhance EV yield, processing efficiency, and suitability for large‐scale production (Haraszti et al. 2018; Kim et al. 2024; Zhang, Cheng, et al. 2024). However, conventional ultracentrifugation, immunoaffinity capture, and most precipitation‐based methods remain difficult to adapt for high‐throughput biomarker screening or clinical‐grade manufacturing. Commercial precipitation kits, although convenient for exploratory work, may introduce polymer carryover or enrich protein and lipoprotein contaminants, making them poorly suited for applications that require stringent purity, potency, and batch‐to‐batch consistency. In the context of therapeutic translation, scalability encompasses considerations in product development. EV‐based products must demonstrate controlled source material, consistent manufacturing, impurity profiling, validated potency assays, defined dose metrics, storage stability, established release criteria, biodistribution assessment, and nonclinical safety evaluation before claims of efficacy or platform‐level advantages (Takakura et al. 2024; Terai et al. 2025; Verma and Arora 2025; Xu, Jin, et al. 2025).

Biological heterogeneity presents significant challenges for data interpretation. EV yield and cargo composition vary across cell types, oncogenic status, hypoxic conditions, inflammation, metabolic stress, treatment exposure, and culture conditions. In studies utilizing CM, this heterogeneity is further influenced by serum depletion protocols, residual serum EV contamination, non‐physiological culture environments, conditioning intervals, cell density, and the lack of systemic host context. Indeed, while CM models offer experimental control, they do not replicate physiological complexity. Their primary advantage is the ability to define and report experimental variables that would otherwise remain unregulated, rather than to eliminate variability entirely (Mukerjee et al. 2025). This limitation is particularly pertinent in tumor EV research, as CM systems do not recapitulate systemic EV dilution and clearance, vascular transport, immune surveillance, organ‐specific uptake, endocrine and metabolic effects, or contributions from host‐derived EVs. Therefore, findings derived from CM are most valuable as mechanistic hypotheses for subsequent validation in biofluids, animal models, and clinically annotated specimens (Shekari et al. 2023; Welsh et al. 2024).

Serum and culture artifacts represent a particularly important subset of these limitations. Residual bovine or serum‐derived EVs can persist in EV‐depleted serum, and heat inactivation performed after EV depletion can alter the proteome and introduce proteins that co‐isolate with cell‐derived EV preparations. These observations reinforce the need to report and interpret serum source, depletion strategy, heat‐inactivation sequence, serum‐free interval, cell viability, and stress responses as experimental variables, not as background details (Pham et al. 2021; Shekari et al. 2023; Urzi et al. 2024).

A second cross‐cutting limitation concerns functional causality. Although many CM studies report recipient‐cell phenotypes after exposure to EV‐enriched fractions, EV‐specific attribution requires comparison with whole CM and EV‐depleted CM, add‐back or rescue designs, cargo perturbation, uptake or release blockade, enzymatic controls with and without membrane disruption when appropriate, and dose‐normalized assays. Without these controls, apparent EV effects may reflect soluble mediators, extracellular ribonucleoprotein complexes, protein aggregates, residual serum components, or preparation‐load differences (Mateescu et al. 2017; Welsh et al. 2024).

These limitations also condition the translation of diagnostics and therapy. For biomarker studies, the main challenge is to demonstrate that an EV‐associated signal provides clinically meaningful information beyond established liquid biopsy or protein marker workflows under standardized pre‐analytical conditions. For therapeutic studies, the corresponding challenge is product‐specific: EV source, engineering strategy, cargo loading, potency, biodistribution, safety, and release criteria must be defined for each platform and cannot be inferred from EV biology as a class (Terai et al. 2025; Welsh et al. 2024; Xu, Jin, et al. 2025).

Taken together, these challenges support several priorities: CM‐aware workflow standardization, higher‐resolution microfluidic and single‐particle technologies, multi‐omics integration with complete metadata, rigorous functional‐causality frameworks, and regulatory‐grade criteria for EV‐based diagnostics and therapeutics (Shekari et al. 2023; Welsh et al. 2024; Xu, Jin, et al. 2025).

## 7 Future Directions

Future work needs to move beyond general enthusiasm for EV methodologies and toward CM‐aware EV workflows that explicitly connect culture design, EV enrichment, characterization, functional causality, and translational feasibility. The most useful advances will be those that improve analytical resolution while also reducing sample loss, pre‐analytical variability, co‐isolate burden, and ambiguity in EV subtype assignment. In this context, future directions are most valuable when they strengthen the evidential chain from CM generation to EV‐specific mechanisms and to clinical or therapeutic translation, rather than when they merely add another isolation or profiling technology (Shekari et al. 2023; Welsh et al. 2024).

A first priority is to make CM models more interpretable, not merely more productive. Future studies need to treat donor‐cell identity, serum strategy, culture dimensionality, oxygen tension, conditioning interval, cell viability, mycoplasma status, CM handling, and storage as experimental variables that can alter EV yield, cargo composition, co‐isolate burden, and biological activity. These variables should be reported alongside EV characterization and functional readouts so that observed effects can be attributed to EV‐enriched fractions, whole CM, soluble mediators, or co‐isolated extracellular material with appropriate caution (Shekari et al. 2023; Welsh et al. 2024).

A second priority is the transition from descriptive EV association to rigorous causal testing. Achieving this objective requires increased use of EV‐depleted CM, add‐back or rescue experimental designs, cargo perturbation, uptake or release inhibition, dose–response analyses, and normalization strategies that differentiate particle number, EV‐associated proteins, lipid content, and donor‐cell‐equivalent input. For RNA‐cargo claims, functional interpretation should also require evidence of cargo protection or accessibility, recipient‐cell uptake, and biological effects that depend on the perturbation (Mateescu et al. 2017; Welsh et al. 2024).

A third priority is to integrate technological innovation with the translational discipline. Microfluidic, nanotechnology‐based, single‐vesicle, and multi‐omics workflows require evaluation not only for sensitivity or throughput but also for recovery, capture bias, EV specificity, co‐isolate control, metadata completeness, cross‐platform reproducibility, and compatibility with downstream functional assays. In parallel, EV‐based diagnostic and therapeutic development remains product‐ or biomarker‐specific, with attention to analytical validation, clinically meaningful thresholds, source control, potency assays, biodistribution, safety, scalable production, and release criteria (Takakura et al. 2024; Welsh et al. 2024; Xu, Jin, et al. 2025).

The following subsections address three interrelated areas: microfluidic and nanotechnology‐based EV isolation and analysis; multi‐omics integration for cargo discovery and pathway reconstruction; and the clinical translation of EV diagnostics and therapeutics within comparator‐aware, regulatory‐grade development frameworks. These technologies are not inherently decisive but are valuable for reducing uncertainty in CM‐derived EV research and for enabling more reproducible, mechanistically grounded, and clinically relevant outcomes.

### 7.1 Microfluidic and Nanotechnology‐Based Isolation

Microfluidic platforms have the potential to enhance CM‐derived tumor EV workflows by integrating EV enrichment, capture, and analysis within miniaturized systems that require minimal input volumes (Dai et al. 2025; Woo et al. 2025). Techniques such as deterministic lateral displacement, dielectrophoresis, acoustic fractionation, immunocapture, and hybrid microfluidic strategies enable the separation of EV‐enriched fractions based on size, physical properties, or surface phenotype (Calero et al. 2025; Woo et al. 2025). For CM‐derived tumor EV research, the critical performance criteria are input volume, recovery, purity, capture bias, vesicle integrity, co‐isolate reporting, and compatibility with downstream RNA, protein, single‐particle, and functional assays (Welsh et al. 2024). Given that cell‐culture conditions and CM handling can alter EV yield and cargo, microfluidic performance needs to be interpreted in relation to donor‐cell metadata, culture format, conditioning interval, and pre‐analytical handling (Shekari et al. 2023).

Proof‐of‐concept platforms illustrate both the technical promise and the need for cautious interpretation. Han et al. developed a lipid nanoprobe‐modified microvortex chip using Morpho menelaus butterfly wings to enhance vesicle capture through lipid‐bilayer affinity, and the platform supported downstream RNA extraction (Han et al. 2020). Guo et al. introduced a paper‐based isotachophoresis system integrated with lateral‐flow detection, enabling rapid multiplexed EV profiling in that experimental setting (Guo, Xu, et al. 2020). These examples support analytical innovation, but by themselves, they do not establish universal recovery, purity, or clinical diagnostic performance.

A more CM‐specific example is the three‐dimensional self‐assembled SiO_2_ nanostructured microfluidic chip developed to capture CD63‐ and CD81‐positive EVs from breast cancer‐derived conditioned cell‐culture media (Cabeza et al. 2025). This platform demonstrates the value of antibody‐functionalized microfluidics for marker‐positive EV detection; however, its output reflects selective capture of epitope‐positive EV subsets, not total EV recovery or proof of exosomal origin (Welsh et al. 2024).

Nanoparticle‐assisted and spectroscopy‐based approaches may also improve EV analysis by providing molecular or phenotypic resolution beyond bulk particle counts. Surface‐enhanced Raman spectroscopy has been used for label‐free EV detection and phenotypic discrimination in proof‐of‐concept studies (Parlatan et al. 2023), and SERS‐based EV detection has been reviewed as a candidate cancer‐diagnostic approach with important assay and standardization constraints (Li et al. 2022). Recent reviews of integrated microfluidic EV platforms and on‐chip quantification indicate that the field is moving toward combined separation, detection, molecular profiling, and single‐EV or subpopulation analysis (Calero et al. 2025; Dai et al. 2025; Woo et al. 2025). Analytical gains thus require benchmarking against recovery, dynamic range, calibration, antibody specificity, matrix effects, and cross‐platform reproducibility (Welsh et al. 2024).

In future studies of CM‐derived tumor EVs, the most effective microfluidic or nanotechnology‐based workflows will incorporate cross‐validated EV characterization, co‐isolate assessment, dose normalization, and functional add‐back or rescue designs when biological activity is asserted (Mateescu et al. 2017; Welsh et al. 2024). In the absence of these components, miniaturized capture or sensing platforms may enhance analytical sensitivity but may not improve EV‐specific causal interpretation or clinical applicability.

### 7.2 Integration of Multi‐Omics Approaches

Multi‐omics approaches remain essential for characterizing EV‐associated RNA, protein, lipid, and metabolite cargo. However, in CM‐derived tumor EV studies, these approaches should extend beyond cargo cataloging to include donor‐cell metadata, culture conditions, EV‐enrichment workflows, co‐isolate assessment, and dose‐normalized functional readouts (Shekari et al. 2023; Welsh et al. 2024). This comprehensive approach is necessary because EV cargo profiles are influenced by both tumor biology and pre‐analytical or culture‐related variables (Shekari et al. 2023; Welsh et al. 2024). Furthermore, database‐derived cargo lists serve as discovery resources but do not constitute evidence of EV purity, disease specificity, cellular origin, or functional causality (Chitti et al. 2024; Gummadi et al. 2025; Welsh et al. 2024).

The ExoCarta 2024 update reported data from 1249 small EV studies, including 119,489 protein entries, 15,868 RNA entries, 3946 lipid entries, and quantitative data for 24,073 entries (Gummadi et al. 2025). These figures illustrate the scale of sEV cargo cataloging; however, they should be regarded as discovery resources rather than as validation of individual biomarkers or pathways. Vesiclepedia 2024 provides a broader compendium of EVs and extracellular particles, covering DNA, RNA, proteins, lipids, and metabolites (Chitti et al. 2024). Therefore, the interpretation of downloaded datasets needs to account for sample source, isolation/separation method, EV or extracellular‐particle subtype annotation, and study‐level metadata (Chitti et al. 2024; Welsh et al. 2024).

When combined with controlled isolation platforms and complete metadata, integrated proteomic, transcriptomic, lipidomic, metabolomic, and computational workflows can help identify EV‐associated signatures and prioritize mechanistic hypotheses (Liang, Lehrich, et al. 2021; Shaba et al. 2022). For explicitly machine‐learning‐based tumor‐EV discovery or classification, recent EV/ML tumor‐diagnosis literature provides more directly relevant support than non‐EV‐specific machine‐learning references (Xu, Li, and Gong 2025). Candidate signatures require independent cohorts, technically harmonized EV workflows, transparent model validation, and multiparametric readouts before they can be treated as biomarkers or mechanisms (Welsh et al. 2024; Xu, Li, and Gong 2025). Single‐EV or subpopulation‐resolved platforms are particularly important for liquid‐biopsy applications because rare tumor‐derived EVs may be masked by the larger background of host‐derived vesicles (Imanbekova et al. 2025).

For CM‐derived systems, the most informative multi‐omics studies will connect cargo discovery to functional causality through donor‐cell perturbation, EV‐cargo perturbation, recipient‐cell readouts, add‐back or rescue experiments, and dose‐normalized comparisons across culture conditions (Mateescu et al. 2017; Shekari et al. 2023; Welsh et al. 2024). Under this framework, multi‐omics becomes a mechanism‐generating and validation‐guiding strategy rather than a stand‐alone catalog of EV‐associated molecules.

### 7.3 Clinical Translation and Therapeutic Innovation

EV‐based drug‐delivery platforms remain promising, although the current evidence does not support their universal superiority over synthetic lipid nanoparticles or other nanocarriers (Bader et al. 2024). Their relative advantages in biocompatibility, immunogenicity, biodistribution, targeting, and cargo protection are likely to depend on EV source, engineering strategy, cargo type, manufacturing process, disease indication, and comparator platform (Agnihotram et al. 2024; Takakura et al. 2024; Xu, Jin, et al. 2025). Selective cargo loading, scalable production, purification, storage stability, potency assays, release criteria, and reproducible batch characterization remain core translational barriers (Takakura et al. 2024; Xu, Jin, et al. 2025). Therefore, a comparator‐aware roadmap requires specification of the reference delivery system, the matched payload, the route, the dose, the schedule, and aligned efficacy and safety endpoints before therapeutic superiority is inferred (Bader et al. 2024; Xu, Jin, et al. 2025).

Recent early clinical experience with engineered exosomes carrying KRAS^G12D^‐specific siRNA in pancreatic cancer provides the strongest current clinical anchor for EV‐based oncogene‐targeted delivery (Kalluri et al. 2025). The iEXPLORE Phase I study is best described as an early‐phase safety, tolerability, target engagement, and immunological correlative signal in a defined product and indication, rather than as evidence of broad efficacy or platform‐level superiority over non‐EV RNA‐delivery systems (Bader et al. 2024; Kalluri et al. 2025). Its main value to the field is demonstrating that selected engineered EV products can enter first‐in‐human testing when supported by product‐specific manufacturing, pharmacology, toxicology, and mechanism‐linked readouts (Kalluri et al. 2025; Xu, Jin, et al. 2025). Definitive clinical benefit, optimal dose, repeat‐dosing feasibility, and comparative performance remain open questions.

Accordingly, the next stage of clinical translation requires prioritizing product‐specific development frameworks that connect EV source, engineering strategy, cargo loading, purity, impurity profile, potency assay, biodistribution, pharmacodynamic readout, safety package, storage stability, and release criteria to the intended indication (Takakura et al. 2024; Terai et al. 2025; Xu, Jin, et al. 2025). For oncology, CM‐based mechanistic models require integration with biofluid‐derived EV studies, orthotopic or patient‐derived in vivo validation, and clinical samples, as no single system is sufficient to establish therapeutic relevance (Shekari et al. 2023; Welsh et al. 2024). Regulatory‐facing studies need to define critical quality attributes, manufacturing‐process controls, assay acceptance criteria, and comparator platforms early, given that EV products remain an evolving therapeutic class (Takakura et al. 2024; Terai et al. 2025; Xu, Jin, et al. 2025).

Progress will therefore depend not only on improved isolation technologies but also on harmonized reporting and independent complementary characterization, validated functional controls and dose‐normalized assays (Mateescu et al. 2017; Welsh et al. 2024), transparent metadata for CM‐derived systems (Shekari et al. 2023), and interdisciplinary collaboration among EV biologists, engineers, clinicians, data scientists, manufacturing specialists, and regulatory experts. These requirements connect methodological innovation to clinical readiness: CM‐derived tumor EV studies generate mechanistic hypotheses, whereas diagnostic and therapeutic translation requires biomarker‐ or product‐specific validation pathways (Terai et al. 2025; Welsh et al. 2024; Xu, Jin, et al. 2025).

## 8 Conclusion

Conditioned medium‐derived tumor EV research has helped clarify how cancer‐cell‐released EV‐enriched preparations can contribute to oncogenic intercellular communication, including metastasis and pre‐metastatic niche formation (Hoshino et al. 2015; Peinado et al. 2012), immune evasion through checkpoint ligands and immunoregulatory cargo (Kim et al. 2019; Shen et al. 2022; Whiteside 2016), stromal and vascular remodeling (Cho et al. 2012; Fang et al. 2018; Zeng et al. 2018), and therapy resistance through intercellular transfer or therapeutic sequestration of resistance‐associated molecules (Aung et al. 2011; Liu et al. 2017; Steinbichler et al. 2019). Across these studies, the recovered material remains best defined as tumor‐derived EV‐enriched or small EV‐enriched fractions unless exosomal/endosomal origin has been directly established (Thery et al. 2018; Welsh et al. 2024).

CM systems remain valuable because they allow donor‐cell identity, culture conditions, and experimental perturbations to be controlled more explicitly than is usually possible in patient biofluids (Shekari et al. 2023). Their value is methodological, not automatically physiological: serum‐depletion strategy, cell density, culture format, conditioning interval, oxygen tension, handling, and storage can alter EV yield, cargo, and apparent activity (Shekari et al. 2023), while co‐isolated soluble proteins, extracellular ribonucleoprotein particles, protein aggregates, lipoprotein‐like material, and residual serum‐derived components can confound attribution to EVs (Pham et al. 2021; Welsh et al. 2024). Consequently, EV‐specific biological attribution is strongest when EV‐enriched fractions, EV‐depleted CM, add‐back or rescue experiments, cargo perturbation, enzymatic controls, and dose‐normalized functional assays are integrated into the experimental design (Mateescu et al. 2017; Welsh et al. 2024).

The diagnostic potential of extracellular vesicle (EV)‐associated biomarkers is demonstrated by specific miRNA and protein signatures that show promise for cancer detection, subtype discrimination, prognostic assessment, and disease monitoring (De Giorgis et al. 2024; Jin et al. 2017; Pham et al. 2025; Tang et al. 2020; Wong and Chen 2019). However, their clinical application remains limited to candidate or panel‐based approaches and is not yet fully established. This limitation arises because biomarker performance is influenced by factors such as the biofluid source, pre‐analytical variables, EV enrichment strategy, assay platform, tumor‐EV abundance, cut‐off definition, and the need for independent validation (Imanbekova et al. 2025; Shan et al. 2025; Welsh et al. 2024).

Therapeutically, EV biology has generated two distinct translational strategies: targeting tumor‐promoting EV pathways identified through CM and in vivo models (Datta et al. 2017; Welsh et al. 2024; Zhao et al. 2022), and developing engineered EV platforms for the delivery of drugs, RNA, cytokines, vaccines, or immune agonists (Cheng and Kalluri 2023; Jang et al. 2021; Kamerkar et al. 2017). Initial clinical studies with KRASG12D‐targeted engineered exosomes demonstrate feasibility, safety, tolerability, and target engagement in selected patients; however, they do not establish definitive clinical efficacy or superiority over lipid nanoparticles or alternative delivery systems (Bader et al. 2024; Kalluri et al. 2025). Successful clinical translation will therefore depend on product‐specific definition of source material, cargo loading, potency, biodistribution, dose metrics, GMP‐compatible manufacturing, storage stability, release criteria, and nonclinical safety (Takakura et al. 2024; Terai et al. 2025; Xu, Jin, et al. 2025).

Overall, CM‐derived tumor EV studies provide a powerful but operationally constrained framework for mechanistic oncology research. The primary value of these studies lies not in their ability to fully replicate systemic cancer biology but in their capacity to define vesicle‐associated mechanisms, culture variables, co‐isolates, and functional controls with exceptional experimental precision (Shekari et al. 2023; Welsh et al. 2024). Advancement in this field will require the integration of CM‐based mechanistic investigations with analyses of biofluid‐derived EVs, in vivo validation, single‐vesicle and multi‐omics approaches, and endpoints that are both clinically relevant and suitable for regulatory evaluation. Within this hierarchy of evidence, tumor‐derived EVs continue to provide important biological insights and translational opportunities; however, diagnostic and therapeutic interpretations should remain specific to the EV type, workflow, and validation status.

## Author Contributions

**Alaya Alkaabi:** data curation (equal), visualization (equal), writing – original draft (equal). **Dana Nasrallah:** data curation (equal), visualization (equal), writing – original draft (equal). **Roberta Giordo:** methodology (equal), visualization (equal), writing – review and editing (equal). **Gheyath K. Nasrallah:** resources (equal), visualization (equal), writing – review and editing (equal). **Hatem Zayed:** project administration (equal), resources (equal), supervision (equal). **Gianfranco Pintus:** conceptualization (lead), funding acquisition (lead), project administration (equal), resources (equal), supervision (equal), writing – review and editing (equal).

## Funding

This work has been developed within the framework of the eINS‐Ecosystem of Innovation for Next Generation Sardinia (cod. ECS 00000038), a project funded by the Italian Ministry of University and Research (MUR) under the National Recovery and Resilience Plan (PNRR). This work was also made possible thanks to “Progetto Fondazione di Sardegna‐Bando 2022–2023” and “DM 737/2021 resources 2022–2023, funded by the European Union—NextGenerationEU”.

## Disclosure

Declaration on the use of AI‐assisted and graphical tools: During the preparation of this manuscript, AI‐assisted language‐editing tools were used only for limited language refinement, grammatical editing, and readability improvement. FigureLab was used as an AI‐assisted graphical tool to create original icons for the schematic figures. BioRender and Microsoft PowerPoint were subsequently used for final graphical layout, editing, and formatting. AI‐assisted tools were not used to generate scientific content, select or interpret the literature, create hypotheses, formulate conclusions, verify references, or create unsupported scientific claims. All figures are original conceptual illustrations conceived, reviewed, and finalized by the authors. All scientific content, references, interpretations, figures, and final text were critically reviewed, verified, and approved by the authors, who take full responsibility for the integrity, accuracy, and originality of the manuscript.

## Ethics Statement

The authors have nothing to report.

## Consent

The authors have nothing to report.

## Conflicts of Interest

The authors declare no conflicts of interest.

## Related WIREs Articles

A systematic approach to exosome‐based translational nanomedicine.

Engineered Macrophage Exosomes Deliver Drug‐Targeted Therapy for Breast Cancer.

## Data Availability

Data sharing does not apply to this article as no new data were created or analyzed in this study.
